# Rotational Thromboelastometry (ROTEM) Hemostasis Profile in Pregnant Women with Preeclampsia and Their Offspring: An Observational Study

**DOI:** 10.3390/diagnostics15172156

**Published:** 2025-08-26

**Authors:** Christos-Georgios Kontovazainitis, Dimitra Gialamprinou, Alexandra Fleva, Theodoros Theodoridis, Ilias Chatziioannidis, Christina Mitsiakou, Anastasia Banti, Elissavet Diamanti, Georgios Mitsiakos

**Affiliations:** 1 2nd Neonatal Department and Neonatal Intensive Care Unit (NICU), Papageorgiou University Hospital, Aristotle University of Thessaloniki, 56403 Thessaloniki, Greece; chgkontovazainitis@gmail.com (C.-G.K.); gialamprinou@gmail.com (D.G.); diamanti.veta@gmail.com (E.D.); 2 Immunology and Histocompatibility Department, Papageorgiou University Hospital, Aristotle University of Thessaloniki, 56403 Thessaloniki, Greece; alexfleva@gmail.com; 3 1st Department of Obstetrics and Gynecology, Papageorgiou University Hospital, Aristotle University of Thessaloniki, 56403 Thessaloniki, Greece; theodtheo@yahoo.gr; 4 1st Neonatal Department and Neonatal Intensive Care Unit (NICU), Ippokrateio University Hospital, Aristotle University of Thessaloniki, 54642 Thessaloniki, Greece; drilias@windowslive.com; 5 2nd Department of Paediatrics, AHEPA University Hospital, Aristotle University of Thessaloniki, 54636 Thessaloniki, Greece; christina.mits@outlook.com; 6 Haematology Department, Papageorgiou University Hospital, Aristotle University of Thessaloniki, 56403 Thessaloniki, Greece; anastasia_mpanti@yahoo.com

**Keywords:** pregnancy, pre-eclampsia, hypertension, pregnancy induced, hemostasis, viscoelastic tests, thromboelastometry, ROTEM, infant, newborn

## Abstract

**Background/Objectives**: Conventional Coagulation Tests (CCTs) fail to accurately reflect Preeclampsia’s (PE’s) coagulation status, disease progression, and hemostatic alterations. They do not differentiate between the normal hypercoagulability of healthy pregnancies and the pathological hypercoagulability associated with PE. Rotational Thromboelastometry (ROTEM) analyzes clot dynamics from initiation through amplification and propagation to termination and fibrinolysis. However, their application in PE, particularly in neonates born to women with PE, is limited. We aimed to identify the hemostatic alterations in pregnant women with PE using ROTEMs that remain undetected by CCTs and to assess PE’s impact on neonatal hemostasis at birth. **Methods**: This was a single-center observational study (March 2022–March 2024) including 31 women with PE (34 newborns) and 45 pregnancies without PE (47 newborns). Maternal blood was collected intrapartum before placental delivery. Neonatal arterial samples were obtained within the first hour of life before vitamin K administration. ROTEM (Intrinsic (INTEM), Extrinsic (EXTEM), Fibrinogen (FIBTEM), Aprotinin (APTEM)), and CCTs were performed. Subgroup analyses considered PE severity and onset. ROC analyses examined discrimination for persistent maternal thrombocytopenia within 7 days of delivery and association with maternal platelet transfusion. **Results**: In preeclamptic women, the INTEM and FIBTEM assays were more affected, with higher Actual Clot Firmness (ACF) (*p* = 0.03, *p* = 0.04, respectively) and a higher Clot Formation Rate (CFR) (*p* = 0.03, *p* = 0.02, respectively). Hyperfibrinolysis was present (CT-APTEM < CT-EXTEM, MCF-APTEM > MCF-EXTEM). Clot Formation Time CFT-EXTEM was an indicator of maternal platelet transfusion (AUC = 0.81). Across EXTEM, INTEM, and APTEM, A10 (Amplitude at 10 min) and CFT showed good discrimination capability for maternal persistent thrombocytopenia within 7 days of delivery (AUCs 0.82–0.95). Neonates of women with PE presented lower ACF across all assays (INTEM *p* = 0.003; EXTEM *p* = 0.001; FIBTEM *p* = 0.01; APTEM *p* < 0.001), consistent across severity/onset subgroups. **Conclusions**: In this cohort, ROTEM identified maternal hypercoagulability with hyperfibrinolysis and neonatal hypocoagulability during the first hour of life. Several alterations were not reflected in CCTs. Further prospective studies should evaluate the role and clinical utility of combining ROTEM with CCTs for hemostatic monitoring in women with PE and their neonates.

## 1. Introduction

Normal gestation is a procoagulant state, where coagulation and fibrinolysis are intensified but balanced to maintain hemostasis [1,2,3]. The uteroplacental hemostatic balance adjusts according to physiological changes during pregnancy [2,4,5]. Preeclampsia (PE) affects 2–8% of gestations worldwide [5,6]. Notably, abnormal trophoblastic invasion with consequential abnormal implantation characterizes early-onset PE, whereas late-onset PE is thought to be influenced by maternal constitutional factors [7]. In either case, the above pathologies lead to endothelial activation and dysfunction, which is mediated by immunological and inflammatory factors, and is mainly mirrored by excessive hypercoagulability, hypertension, proteinuria, and other clinical manifestations [2,6,8]. The gestational hemostatic balance is vulnerable to PE’s endothelial dysfunction, leading to platelet hyperactivation and hypercoagulation, with elevated Tissue Factor (TF) expression and activity, and perturbed fibrinolysis [2,9].

Conventional coagulation tests (CCTs) have yielded variable and non-concise results in PE, with ambiguous changes in Activated Partial Thromboplastin Time (APTT), often prolonged Prothrombin Time (PT), increased fibrinogen and D-dimers, and decreased antithrombin activity [8,10,11,12,13]. Severe PE cases with platelet counts below 100 × 10^9^/L showed more pronounced alterations, affecting PT, APTT, and fibrinogen [14].

Thus, CCTs fail to depict hemostatic alterations in PE universally [9]. CCTs are conducted on platelet-poor plasma (whereas in vivo clotting occurs on cell surfaces) and cannot account for the dynamic interactions among platelets, coagulation factors, inhibitors, and fibrinolytic proteins [10,13]. As such, CCTs fail to effectively mirror PE’s coagulation status, making it challenging to monitor disease progression. They do not differentiate between the normal hypercoagulability of healthy pregnancies and the pathological hypercoagulability associated with PE, failing to account for the functional quality of the clots.

Viscoelastic tests, namely, Thromboelastography (TEG) and Rotational Thromboelastometry (ROTEM), being quick and easy-to-use bedside methods, are more capable of monitoring the aforementioned PE’s hemostatic alterations. They analyze clot dynamics from initiation through amplification and propagation to termination and fibrinolysis, and are increasingly utilized in obstetrical anesthesiology [15,16,17]. Murray et al. demonstrated that CCTs showed no consistent differences between PE and healthy pregnancies, whereas TEG identified a hypercoagulable state in PE. Such data indicate that clinicians cannot rely on CCTs to estimate PE-associated coagulopathy, highlighting the need for more sensitive, whole-blood assays [10]. The application of viscoelastic tests in PE, particularly in neonates born to women with PE, is limited, and the literature is sparse [14,18,19].

Clinicians have to make management decisions (such as anesthesia plans or readiness for hemorrhage) without reliable coagulation indicators. This is especially problematic in severe PE or HELLP syndrome—scenarios where detection of coagulopathy is critical. Indeed, case reports note that viscoelastic tests have helped diagnose and treat hemorrhagic complications in HELLP syndrome when CCTs were unhelpful [5].

In summary, CCTs do not provide clinicians with reliable information to manage coagulation in PE. There is a critical need for better tools to monitor hemostatic alterations in these patients. Viscoelastic assays (TEG/ROTEM) offer a dynamic assessment of clot quality and have shown promise in detecting the hypercoagulable state of PE when conventional tests fail. This study aims to use ROTEM to identify hemostatic changes in pregnant women with PE that CCTs cannot detect, and to evaluate the neonatal hemostatic profile during the first hour of life using the same method.

## 2. Materials and Methods

### 2.1. Study Design, Setting, and Participants

This one-center observational study compared the hemostatic profiles of pregnant women with PE and their neonates with the hemostatic profiles of pregnant women without PE and their neonates. The study was conducted at “Papageorgiou” Hospital of the Aristotle University of Thessaloniki (Greece) from March 2022 to March 2024. The study adhered to the Helsinki Declaration and was approved by the University’s Department of Bioethics (No. 48/2022) and the Institutional Review Committee (No. 20192/2022).

The study enrolled women who provided informed consent for themselves and their offspring. Eligible participants included pregnant women ≥ 18 years with a Gestational Age (GA) ≥ 20 weeks at enrollment and ≥24 weeks at delivery. Exclusion criteria comprised chronic hypertension before 20 Gestational Weeks (GWs), gestational hypertension, diabetes mellitus, hereditary thrombophilias, antiphospholipid syndrome, systemic lupus erythematosus, other autoimmune/rheumatic diseases, COVID-19 infection (within 15 days before delivery), and clinical chorioamnionitis. Other viral infections were not reasons for exclusion. Women with cancer were excluded since neoplasm-associated coagulopathy could alter the hemostasis results. Women with mental illness and alcoholism were not de facto legally able to sign the informed consent for themselves and their neonates, and, thus, were excluded. Multiple gestations (triplet and above) were excluded. Neonatal inclusion criteria: all neonates born alive to the initially enrolled mothers were initially eligible for inclusion. Those with perinatal asphyxia and pre- or postnatal diagnosis of any chromosomal or other congenital deficiency were excluded. If a neonate was excluded, its mother was also excluded.

Demographic data and medical histories for every mother–neonate pair were recorded from medical records and personal interviews, with monitoring until discharge.

### 2.2. Definitions

Gestational hypertension, PE, PE with severe features, and Hemolysis, Elevated Liver enzymes, and Low Platelet (HELLP) syndrome were defined according to the American College of Obstetrics and Gynecology (ACOG) criteria [6]. More precisely, Gestational Hypertension was defined as blood pressure ≥ 140/90 mmHg on two occasions at least 4 h apart after 20 GWs in a woman with previously normal blood pressure. PE was defined as new-onset hypertension (blood pressure ≥ 140/90 mmHg on two occasions at least 4 h apart in a previously normotensive woman) after 20 GWs, accompanied by either proteinuria or systemic end-organ dysfunction. Proteinuria was defined as ≥300 mg of protein in a 24-h urine collection. End-organ dysfunction was defined as at least one of the following: thrombocytopenia (Platelet (PLT) count < 150 × 10^9^/L), renal insufficiency (serum creatinine > 1.1 mg/dL or a doubling of the baseline value), impaired liver function (elevated liver transaminases to ≥2 times the upper normal limit), pulmonary edema, or new-onset cerebral or visual disturbances. Severe PE was defined as the presence of any of these severe features, including severe hypertension (blood pressure ≥ 160/110 mmHg) or the aforementioned organ complications. The National Institute for Health and Care Excellence (NICE) guidelines were also considered: we used the presence of uteroplacental dysfunction as a diagnostic criterion (fetal growth restriction, abnormal umbilical artery Doppler findings in the presence of hypertension), and thrombocytopenia was defined as a PLT count < 150 × 10^9^ [20]. Early-onset PE was defined as PE occurring before 34 GW [7,21,22]. HELLP syndrome was defined as lactate dehydrogenase (LDH) ≥ 600 IU/L, aspartate aminotransferase (AST), alanine aminotransferase (ALT) ≥ 2 times the upper normal limit, and PLT count < 100 × 10^9^. Chronic hypertension was defined based on the ACOG’s criteria (hypertension diagnosed or present before pregnancy or before 20 weeks of gestation) [23].

### 2.3. Samples and Outcomes

Maternal samples consisted of venous whole blood, while neonatal samples consisted of arterial blood. They were drawn into K3-EDTA (Tripotassium Ethylene-Diamine-Tetraacetic-Acid), serum separator, and trisodium citrate (0.109 mol/L) tubes and were processed within 30 min. Maternal samples were drawn during labor and before the placenta’s delivery. Neonatal samples were drawn within the first hour of life after birth, before vitamin K administration. Neonatal arterial blood is an ideal choice; it requires no venipuncture tourniquet for collection, ensuring an unobstructed blood flow and thus limiting hemostasis activation. Other studies have used cord blood, which can influence the reliability of hemostatic testing due to venous stasis, altering coagulation parameters, even if the sample is drawn shortly after cord clamping.

Whole blood parameters and biochemical parameters were measured using “Alinity-hq” and “Alinity-c” analyzers, respectively (Abbott-GmbH, Wiesbaden, Germany).

CCTs were measured in platelet-poor plasma after centrifugation (1750 G) for 15 min. PT, APTT, Fibrinogen, D-dimers, Antithrombin III, Protein C, and Protein S were assessed using STA-Neoplastine-R15-Plus, STA-Cephascreen-10, STA-Liquid-Fib, STA-Liatest-DDi-Plus, STA-Stachrom-ATIII, STA-Stachrom-ProteinC, and STA-Liatest-FreeProteinS reagents, respectively, using a “STAR-MAX” analyzer (Diagnostica-Stago-S.A.S., Asniers-sur-Seine, France).

The following ROTEM assays were conducted for at least 60 min: Intrinsic, Extrinsic, Fibrinogen, and Aprotinin ROTEM (INTEM, EXTEM, FIBTEM, APTEM, respectively). In the case of Low-Molecular-Weight Heparin administration, Heparinase ROTEM (HEPTEM) was used instead of INTEM. In the INTEM assay, clot formation was induced by activation of the intrinsic coagulation pathway using 20 μL of 0.2 mol/L calcium chloride solution (star-TEM reagent) and 20 μL of intrinsic activator (in-TEM reagent; partial thromboplastin phospholipid made of rabbit brain and ellagic acid). The HEPTEM assay is a modified INTEM assay, where heparin is degraded by 20 μL of heparin inactivator (hep-TEM reagent; heparinase I from flavobacteria). In the EXTEM assay, clot formation was induced by activation of the extrinsic coagulation pathway using 20 μL of 0.2 mol/L calcium chloride solution (star-TEM reagent) and 20 μL of extrinsic activator (ex-TEM reagent; recombinant tissue factor and phospholipids). The FIBTEM assay constitutes a modified EXTEM assay, where platelet contribution to clot formation is inhibited with 20 μL of thrombocyte inhibitor (fib-TEM reagent; Cytochalasin D and 0.2 mol/L calcium chloride). The APTEM assay constitutes a modified EXTEM assay, where fibrinolysis is inhibited with 20 μL of fibrinolysis inhibitor (ap-TEM reagent; plasmin-antagonist Aprotinin and 0.2 mol/L calcium chloride). For each essay, 300 μL of blood mixed with 0.109 mol/L trisodium citrate was analyzed using the ROTEM Delta Analyzer (Tem Innovations GmbH, Munich, Germany) after incubation for 5 min at 37 °C. We measured the following parameters in each assay: Clotting Time (CT), Clot Formation Time (CFT), Maximum Clot Firmness (MCF), A-angle, Amplitude at 10 and 30 min after Clotting Time (A10, 30), Maximum Clot Elasticity (MCE), Lysis Index at 60 min after Clotting Time (LI60), Maximum Lysis (ML), Clot Formation Rate (CFR), and Actual Clot Firmness (ACF). Supplementary Table S1 summarizes the parameters we measured and their interpretation.

### 2.4. Addressing Bias

Selection bias was addressed by recruiting participants for the PE and control groups who were matched by GA, maternal age, drug administration, and other comorbidities. Information bias was addressed by strictly defining the PE criteria and having the same researchers systematically collect both groups’ medical history and outcome data. The same sufficiently trained researchers handled samples. Confounding bias was addressed through nearest-neighbor matching, utilizing the propensity score difference as a distance measure, and by applying p-adjustment methods to control for possible confounders, despite no differences in neonates’ baseline characteristics. Missing data (4.2%) were deleted since they were “missing completely at random”.

### 2.5. Sample Size

We intended to achieve a power of 0.8 with an α error of 0.05 using a two-sided method for computing sample sizes. Regarding pregnant women, based on our previous study [24], the detection of a PLT difference of 71.8 × 10^9^/L (SD = 65.5 × 10^9^/L) required 14 participants per group, while a MCF-FIBTEM difference of 7.6 mm (SD = 9.9 mm) required 28 participants per group. Regarding neonates, based on our previous study [25], to detect a PLT difference of 99.9 × 10^9^/L (SD = 95.6 × 10^9^/L), we needed 15 participants per group, while to detect a MCF-FIBTEM difference of 5.8 mm (SD = 6.7 mm), we needed 22 participants per group. Thus, the sample size of 30 participants in each group was feasible and adequate.

### 2.6. Statistical Analysis

Statistical analysis was conducted using R-Studio (2024.04.2+764, https://dailies.rstudio.com/version/2024.04.2+764/, access date 5 June 2024). Statistical significance was set at α = 0.05.

Regarding two-group comparisons, the two-sample T-test was conducted on quantitative data following a normal distribution, while Welch’s test was used when the assumption of homogeneity of variances was not met. The Mann–Whitney U test was performed for non-normally distributed data.

Regarding three-group comparisons (severity and onset analysis), Fisher’s ANOVA was conducted for normally distributed data. If the *p*-value was ≤0.05 across groups, Tukey’s post hoc analysis was used. The Kruskal–Wallis test was performed for non-normally distributed data. If the *p*-value was ≤0.05 across groups, Dunn’s post hoc analysis was used.

Regarding categorical variables, the Chi-Square test was used. Fisher’s exact test was conducted when the expected numbers were very small. In three-group categorical comparisons (severity and onset analysis) and when *p* ≤ 0.05 across the groups, pairwise comparisons with Bonferroni correction were conducted.

Regarding the neonates’ results, we conducted a one-way ANCOVA (for parametric data) or Quade’s ANCOVA (for non-parametric data) to control for confounding covariates. As confounding covariates, we selected GA (despite no significant differences being recorded) and any baseline characteristics that differed in all analyses.

Correlation analysis was performed using Spearman’s rank correlation coefficient. $\left|\rho \right|$ ≥ 0.6 indicated at least moderate correlations.

## 3. Results

**Figure 1:**
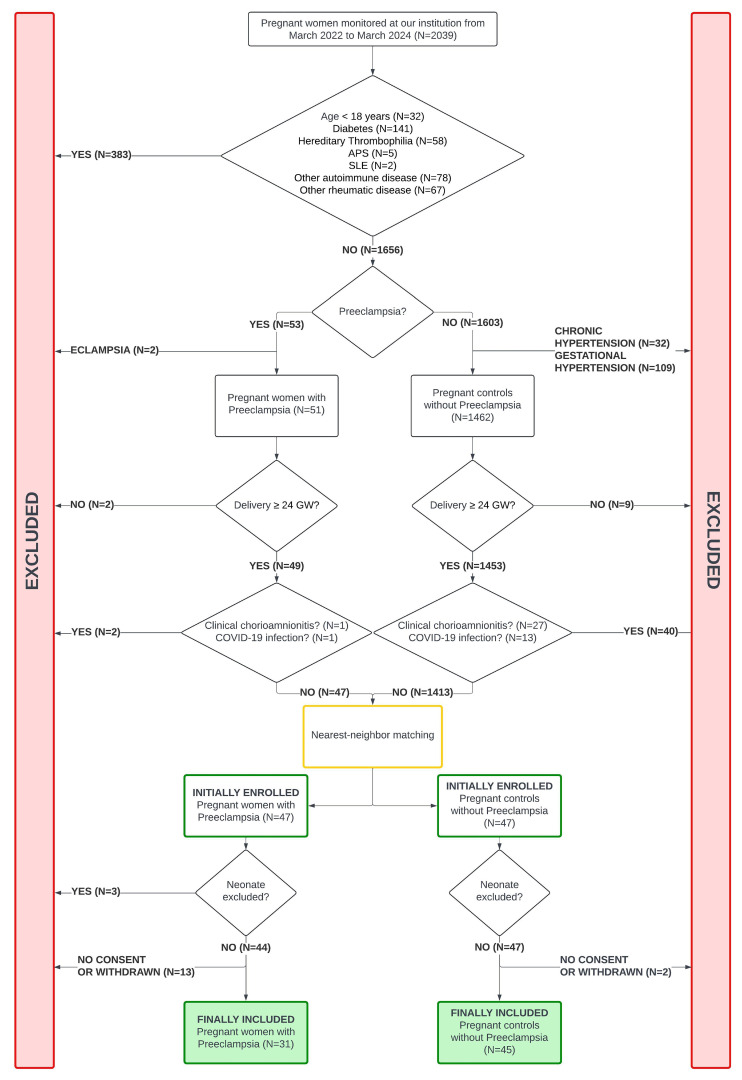
Population diagram (pregnant women). Abbreviations. APS: Antiphospholipid Syndrome; SLE: Systemic Lupus Erythematosus; GW: Gestational Weeks; COVID-19: Coronavirus Disease 2019; PE: Preeclampsia.

**Figure 2:**
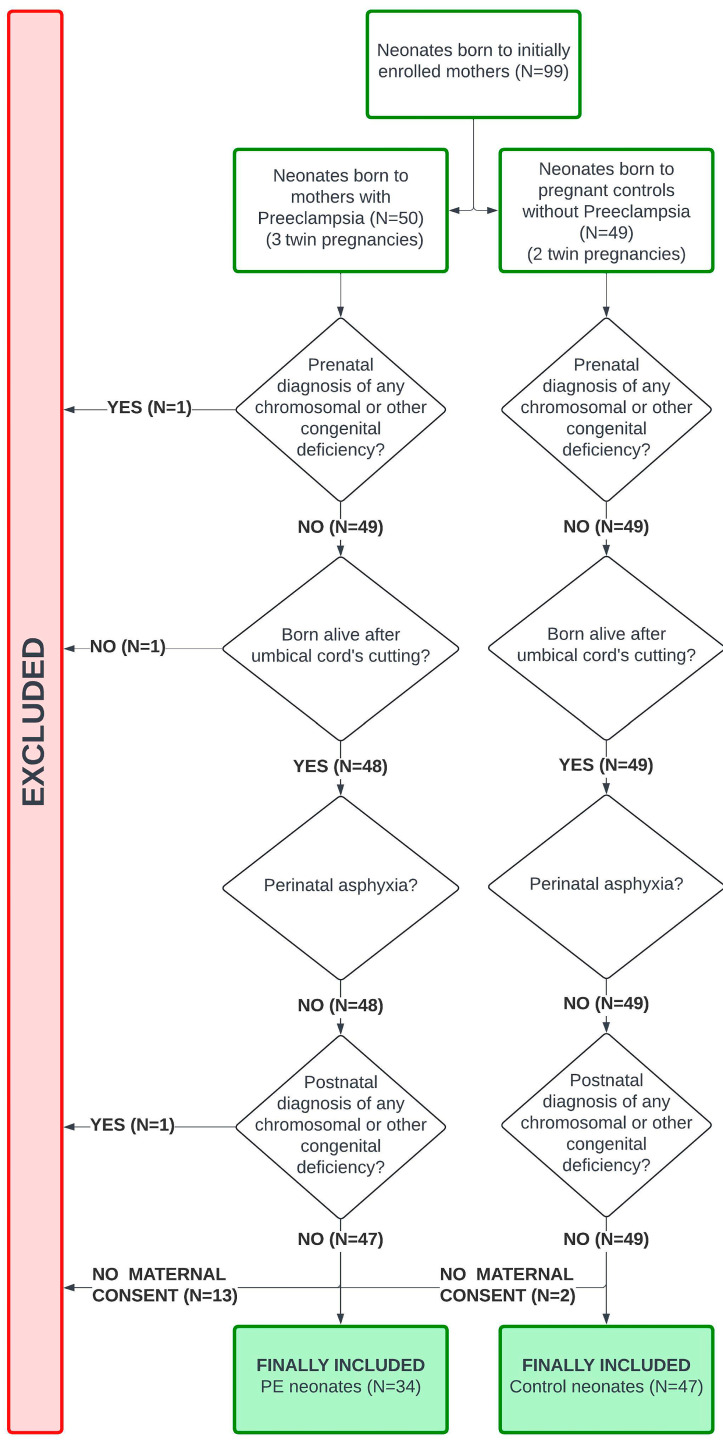
Population diagram (neonates). Abbreviations. PE: Preeclampsia.

A total of 31 mothers with PE and their 34 neonates (three twin pregnancies), and 45 pregnant controls and their 47 neonates (two twin pregnancies) were included (Figure 1 and Figure 2).

### 3.1. Analysis of PE Groups

Regarding pregnant women, no statistically significant differences were found in baseline characteristics between the two groups, except for parity and rates of previous pregnancy loss. No statistically significant differences in baseline characteristics were found between the two groups for neonates, except for the mean uterine artery pulse index and Apgar Score at 5 min (Table 1). Supplementary Table S2 presents the results of maternal and neonatal blood counts and biochemical tests.

Mothers with PE presented with lower PLTs, Free Protein S, and higher D-dimer levels (*p* < 0.001 in all cases). Fibrinogen levels were higher among mothers with PE (*p* = 0.03). The thrombocytopenia rates of mothers with PE (PLT < 150 × 10^9^/L) were higher compared with controls (38.7% vs. 2.2%, respectively, *p* < 0.001). Regarding ROTEM, ML-INTEM was lower, and CFR-INTEM and ACF-INTEM were higher in mothers with PE (*p* = 0.03 in all cases). CT-FIBTEM was lower (*p* = 0.02), and A-angle-FIBTEM, MCE-FIBTEM, CFR-FIBTEM, and ACF-FIBTEM were higher in mothers with PE (*p* = 0.04, 0.04, 0.02, 0.04, respectively). CT-APTEM was lower (*p* = 0.03), and A-angle-APTEM and CFR-APTEM were higher in mothers with PE (*p* = 0.003, 0.001, respectively). Hyperfibrinolysis is present, as evidenced by mothers with PE exhibiting lower CT-APTEM than CT-EXTEM, with higher MCF-APTEM compared with MCF-EXTEM. This pattern is not present among pregnant controls (Table 2).

Neonatal results are adjusted for GA (even if no difference was found between the groups) and Apgar score at 5 min. Neonates born to mothers with PE presented lower PLTs and higher D-dimer levels than controls (*p* = 0.002 and *p* < 0.001, respectively). A total of 15.2% of neonates born to mothers with PE and 2.1% of controls presented with thrombocytopenia at birth. Fibrinogen levels were lower in neonates born to mothers with PE. Regarding ROTEM, CFT was higher in neonates born to mothers with PE in INTEM/EXTEM/APTEM (*p* = 0.005, 0.02, 0.007, respectively), while the A-angle was lower in INTEM/EXTEM (*p* = 0.01, 0.04, respectively). In all assays, A10, A30, MCF, and MCE were lower in neonates born to mothers with PE. LI60 was lower in neonates born to mothers with PE in EXTEM/APTEM (*p* = 0.004, 0.008, respectively), and ML was higher in APTEM (*p* = 0.005). ACF was lower in neonates born to mothers with PE in INTEM/EXTEM/FIBTEM/APTEM (*p* = 0.003, 0.001, 0.01, and <0.001, respectively) (Table 2).

### 3.2. Analysis of PE Severity

Of 31 women, 11 developed severe PE. We matched (propensity score nearest-neighbor matching) the 11 women with severe PE with 11 women with non-severe PE and 19 controls (13, 13, and 21 neonates, respectively).

Regarding pregnant women, no differences in baseline characteristics were found between the subgroups, except for the platelet transfusion rate (Supplementary Table S3). The maternal blood count and biochemical test results are presented in Supplementary Table S4. Maternal PLTs and Free Protein S were significantly lower in the women with severe PE compared with women with non-severe PE and controls. The incidence of thrombocytopenia was significantly higher among the women with severe PE compared with controls. Likewise, the PLT count was lower in women with non-severe PE than in the control group (*p* < 0.001). Fibrinogen levels did not differ across the subgroups, whereas D-dimer levels were significantly higher in both the severe PE and non-severe PE subgroups compared with the control subgroup. Regarding ROTEM, only CT-APTEM was significantly lower in the non-severe subgroup compared with the control subgroup (Supplementary Table S5).

Regarding neonates, no differences in baseline characteristics were found between the subgroups (Supplementary Table S6). The neonatal blood count and biochemical test results are presented in Supplementary Table S7. Neonatal results are adjusted for GA, even if no difference was found between the groups. All neonates born to women with severe PE presented lower PLTs compared with controls (*p* = 0.004). Fibrinogen levels did not differ across the subgroups but were lower in the severe PE subgroup. D-dimer levels were elevated both in the severe and non-severe PE subgroups compared with controls (*p* = 0.003 and *p* = 0.03, respectively). No differences in ROTEM parameters were found between the severe and non-severe PE subgroups. Neonates born to women with non-severe PE presented significantly lower A30-FIBTEM (*p* = 0.048) and significantly lower MCF, A10, A30, MCE, and ACF-APTEM compared with controls. Compared with controls, the neonates born to women with severe PE presented higher CFT in INTEM/APTEM (*p* = 0.001 and *p* = 0.02, respectively), a significantly lower A-angle in INTEM (*p* = 0.005), significantly lower MCF, A10, A30, MCE, and ACF in all assays, lower LI60 in EXTEM/APTEM (*p* = 0.004 and *p* = 0.003, respectively), and higher ML APTEM (*p* = 0.004) (Table 3).

### 3.3. Onset Analysis

Of the 31 women, 19 had early-onset PE (21 neonates) and 12 had late-onset PE (13 neonates). We matched the above women with 32 pregnant controls (34 neonates). Given the de facto definitions of early-onset and late-onset PE, the GA parameter was not considered during the matching process for this analysis.

Regarding pregnant women, no differences in baseline characteristics were found between the subgroups (Supplementary Table S8). Maternal blood count and biochemical test results are presented in Supplementary Table S9. Pregnant women with early-onset PE presented lower PLTs and higher rates of thrombocytopenia (47.4%) compared with controls (*p* < 0.001 in both cases). D-dimer levels were elevated in both the early-onset and the late-onset PE subgroups compared with controls (*p* = 0.002 and *p* = 0.04, respectively). D-dimers were higher in the early-onset PE subgroup than in the late-onset PE subgroup. Fibrinogen was higher in the late-onset subgroup compared with controls (*p* = 0.04). Antithrombin III levels were lower in mothers with early-onset PE than those with late-onset PE (*p* = 0.03). Free Protein S was lower in both the early-onset and late-onset subgroups than in controls (*p* < 0.001 in both cases). Regarding ROTEM, women with early-onset PE presented with lower CT-EXTEM values than those with late-onset PE (*p* = 0.04) and higher CFR-FIBTEM values than controls (*p* = 0.03). Compared with the controls, women with late-onset PE presented higher ACF-INTEM (*p* = 0.02), a higher A-angle FIBTEM/APTEM (*p* = 0.03 in both cases), and higher CFR in FIBTEM/APTEM (*p* = 0.03 and *p* = 0.004, respectively) (Supplementary Table S10).

Regarding neonates, we noted imbalances in baseline characteristics, which came as no surprise, given the de facto definitions of early-onset and late-onset PE (Supplementary Table S11). Neonatal blood count and biochemical test results are presented in Supplementary Table S12. Neonatal results are adjusted for Gestational Age, birthweight, length, head circumference, and Apgar score at 1 and 5 min. Neonates born to mothers with early-onset PE had lower PLTs (*p* = 0.009) and MPV (*p* = 0.04) compared with controls. D-dimer levels were higher in the early- and late-onset PE subgroups compared with controls (*p* = 0.002 and *p* = 0.02, respectively). Fibrinogen levels did not differ across the subgroups but were lower in the early-onset PE subgroup. Compared with controls, neonates born to women with early-onset PE had higher CFT in INTEM/EXTEM (*p* = 0.002 and *p* = 0.02, respectively), and significantly lower MCF, A10, A30, MCE, and ACF in all assays. Compared with the late-onset PE subgroup, neonates in the early-onset PE subgroup had significantly higher CFT and significantly lower MCF, A10, A30, and ACF in INTEM, and significantly lower MCE in INTEM/EXTEM/APTEM. No differences regarding ROTEM were found between neonates in the late-onset PE subgroup and controls (Supplementary Table S13).

### 3.4. Correlations, Regressions, and ROCs

**Figure 3:**
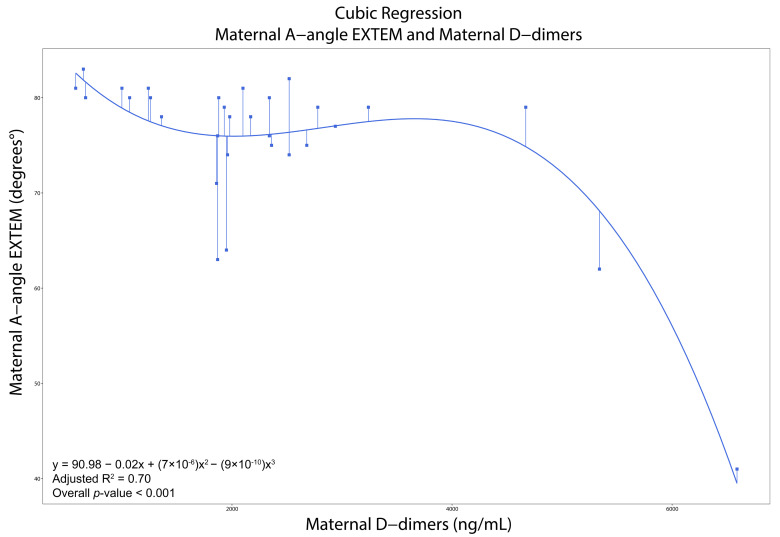
Regression models showing strong and very strong correlations for pregnant women with Preeclampsia. The selection of the model was based on the statistical significance of the *p*-values of the intercept and the coefficients, and the adjusted R^2^. We present those models with adjusted R^2^ ≥ 0.50. Cubic regression with maternal A-angle EXTEM as the dependent variable and maternal d-dimers as the independent variable. Abbreviations. EXTEM: Extrinsic Rotational Thromboelastometry.

**Figure 4:**
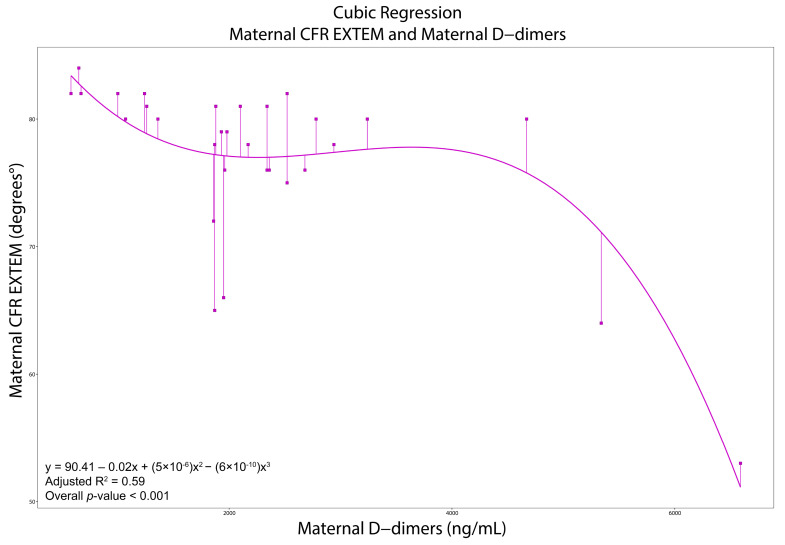
Cubic regression with maternal CFR EXTEM as the dependent variable and maternal d-dimers as the independent variable. Abbreviations. CFR: Clot Formation Rate; EXTEM: Extrinsic Rotational Thromboelastometry.

**Figure 5:**
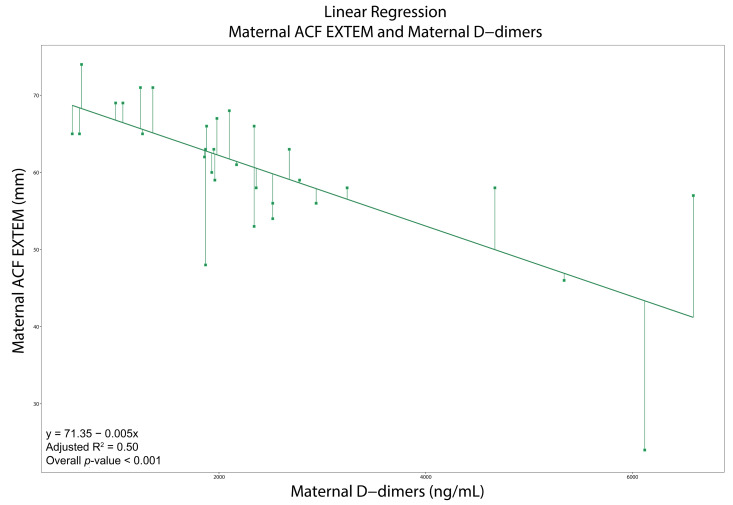
Regression models showing strong and very strong correlations for pregnant women with Preeclampsia. The selection of the model was based on the statistical significance of the *p*-values of the intercept and the coefficients, and the adjusted R^2^. We present those models with adjusted R^2^ ≥ 0.50. Linear regression with maternal ACF EXTEM as the dependent variable and maternal d-dimers as the independent variable. Abbreviations. ACF: Actual Clot Firmness; EXTEM: Extrinsic Rotational Thromboelastometry.

All moderate and strong correlations are presented in Supplementary Tables S14 and S15. In women with PE, LI30-APTEM was negatively correlated with PLTs (*ρ* = −0.65, *p* < 0.001), while A-angle-INTEM was negatively correlated with D-dimers (*ρ* = −0.60, *p* < 0.001). CT-EXTEM was positively correlated with D-dimers (*ρ* = 0.71, *p* < 0.001). Likewise, MCF-EXTEM (*ρ* = −0.68), A-angle-EXTEM (*ρ* = −0.68), A30-EXTEM (*ρ* = −0.70), MCE-EXTEM (*ρ* = −0.66), CFR-EXTEM (−0.67), and ACF-EXTEM (−0.72) were negatively correlated with D-dimers (*p* < 0.001 in all cases). The cubic regression models for maternal A-angle-EXTEM and CFR-EXTEM with maternal D-dimers demonstrated a good fit (R^2^ = 0.70 and 0.59, respectively). The linear regression model for maternal ACF-EXTEM with D-dimers demonstrated a moderate fit (R^2^ = 0.50) (Supplementary Table S16 and Figure 3, Figure 4 and Figure 5).

**Figure 6:**
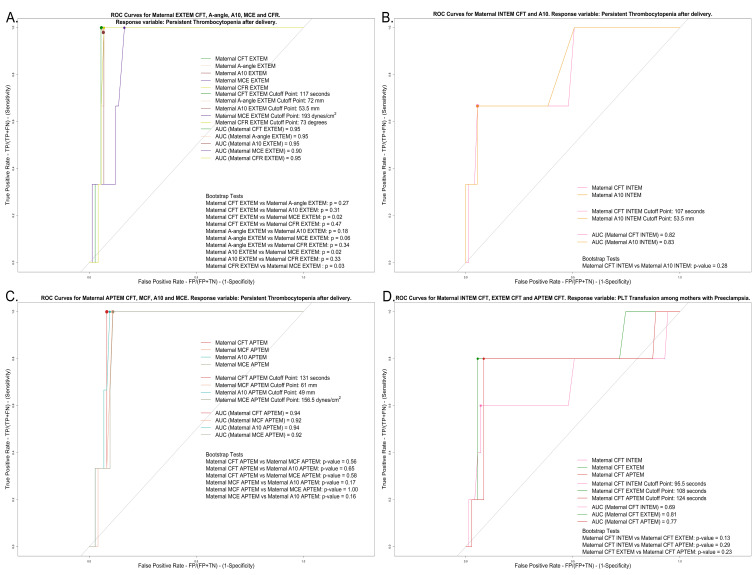
Receiver operating characteristic (ROC) curves for (**A**) maternal EXTEM parameters, with persistent thrombocytopenia up to 7 days after delivery as the response variable; (**B**) maternal INTEM parameters, with persistent thrombocytopenia up to 7 days after delivery as the response variable; (**C**) maternal APTEM parameters, with persistent thrombocytopenia up to 7 days after delivery as the response variable; (**D**) maternal ROTEM parameters, with platelet transfusion at delivery or up to 7 days after delivery as the response variable. Abbreviations. EXTEM: Extrinsic Thromboelastometry; CFT: Clot Formation Time; A10: Amplitude at 10 min post Clotting Time; MCE: Maximum Clot Elasticity; CFR: Clot Formation Rate; INTEM: Intrinsic Thromboelastometry; APTEM: Aprotinin Thromboelastometry; MCF: Maximum Clot Firmness; PLT: Platelet.

Maternal thrombocytopenia (PLT < 150 × 10^9^/L) at delivery was positively associated with CFT-INTEM, LI60-INTEM, and CFT-EXTEM, and negatively associated with A-angle-INTEM, ML-INTEM, MCF-EXTEM, A-angle-EXTEM, A30-EXTEM, MCE-EXTEM, CFR-EXTEM, and ACF-EXTEM. Maternal thrombocytopenia up to 7 days after delivery was positively associated with CFT-INTEM, CFT-EXTEM, and CFT-APTEM, and negatively associated with A10-INTEM, A-angle-EXTEM, A10-EXTEM, MCE-EXTEM, CFR-EXTEM, MCF-APTEM, A10-APTEM, and MCE-APTEM. For all the above, logistic regression models demonstrated a good fit (Supplementary Table S17). Based on the ROC analyses, we noted that A10-INTEM (sensitivity: 0.67; specificity: 0.95), CFT-EXTEM, A-angle-EXTEM, A10-EXTEM, and CFR-EXTEM (sensitivities of 1.00, 0.99, 0,98, and 1.00, respectively; specificities of 0.95, 0.94, 0.93, and 0.93, respectively), and CFT-APTEM and A10-APTEM (sensitivities of 1.00 and 1.00, respectively; specificities of 0.92 and 0.90, respectively) are good predictive factors for persistent thrombocytopenia up to 7 days after delivery. CFT-EXTEM was a good predictive and diagnostic factor for platelet transfusion at delivery and/or up to 7 days after delivery (sensitivity: 0.80; specificity: 0.94) (Figure 6 and Supplementary Table S18).

## 4. Discussion

### 4.1. Main Findings

Among mothers with PE, ROTEM exhibits a hypercoagulable profile, characterized by faster clot initiation, amplification, and propagation phases, as well as increased clot stability, resulting in quicker fibrin formation, thrombin generation, fibrin deposition, and cross-linking. Hyperfibrinolysis is present; on the contrary, ROTEM reveals that neonates born to women with PE show a hypocoagulable profile in the first hour of life compared with those of pregnant controls. Their amplification, propagation phases, and clot stability are affected, slowing thrombin generation, fibrin deposition, and cross-linking.

### 4.2. Pregnant Women

Mothers with PE exhibit elevated fibrinogen and D-dimer levels, indicating enhanced coagulation and fibrinolytic activation, which may potentially reflect inflammation, although C-Reactive Protein (CRP) levels do not confirm this. PLTs were reduced, reflecting platelet activation and consumption. ROTEM demonstrates a hypercoagulable profile in women with PE. The FIBTEM assay, reflecting fibrinogen levels and fibrin polymerization, is notably affected. Mothers with PE exhibit lower CT-APTEM compared with CT-EXTEM, with higher MCF-APTEM compared with MCF-EXTEM, indicating hyperfibrinolysis, which is mirrored by elevated D-dimer levels.

Subgroup analysis based on PE severity reveals that both severe and non-severe PE are associated with PLT activation and consumption, with thrombocytopenia being more frequent in the severe group. D-dimers are elevated in the severe and non-severe PE subgroups, mirroring enhanced fibrinolysis. ROTEM variables do not consistently differentiate these subgroups, likely due to small sample sizes.

There is increased platelet activation and consumption in women with early-onset PE. Women with late-onset PE have a more hypercoagulable profile in ROTEM compared with controls, characterized by faster thrombin generation and increased clot stability. Early-onset PE shows faster initial fibrin formation and enhanced thrombin generation compared with late-onset PE and controls. ROTEM shows enhanced fibrinolysis in early-onset PE through APTEM, EXTEM, and D-dimer levels. Both groups are more hypercoagulable than controls; however, late-onset PE appears to be more hypercoagulable due to higher fibrinogen levels, lower rates of thrombocytopenia, and lower fibrinolysis compared with early-onset PE. If the PLT count falls below a specific limit, a ROTEM hypocoagulable turnover may occur. When the PLT count exceeds this limit, ROTEM parameters seem to plateau, thereby reducing sensitivity to platelet count alterations [5,26,27].

Studies using TEG in pregnant women with PE showed varied results; some indicating hypercoagulability with lower R (Reaction Time—analogous to CT) and K (Clot Kinetics—Analogous to CFT) values and higher A-angle, MA (Maximum Amplitude—analogous to MCF), and CI (Coagulation Index) [8,10], mainly in Non-Severe PE with PLTs > 100 × 10^9^/L. As PE severity increased, MA values dropped significantly compared with controls, especially in cases with critical thrombocytopenia [8,28]. Other studies reported normal R, K, A-angle, MA, and LY60 [9,29], with slight hypocoagulability tendencies [9,28]. Some studies reported a higher K in women with PE, supporting the latter statement [12,28,30]. Since platelet phospholipids play a crucial role in activating factor X and prothrombin, the low PLTs seen in severe PE disrupt the clotting cascade, resulting in extended R and K values. This disruption was further supported by lower MA levels in women with severe PE [9,12,26,30].

Other studies utilizing ROTEM in women with PE showed significant EXTEM alterations, with reduced CT, CFT, and ML, and increased MCF and A-angle, indicating hypercoagulability. This extrinsic pathway imbalance is associated with increased TF, leading to more stable fibrin clots and perturbed fibrinolysis. The increased MCF-FIBTEM and A-angle-FIBTEM along with the strong correlation between MCF-FIBTEM and fibrinogen confirm these findings [11,31]. In HELLP and severe thrombocytopenia, decreased A10-INTEM and A10-EXTEM values reflected disease progression, aligning with hypocoagulability and bleeding risks [32]. Severe thrombocytopenia in PE presented with prolonged CFT-INTEM and CFT-EXTEM, and reduced MCF-INTEM and MCF-EXTEM [31]. Mild thrombocytopenia in PE presented with reduced platelet aggregation and diminished CT-EXTEM, MCF-INTEM, and MCF-EXTEM [31].

Viscoelastic tests in pregnant women generally indicate hypercoagulability until PLTs drop < 100 × 10^9^/L [8,9,10,11,12,26,28,29,30,31,32]. Our regression models confirm this; the EXTEM A-angle, CFR, and ACF remain high and stable with D-dimers < 4000 ng/mL, indicating hypercoagulability. Beyond this threshold (when PLT hyperactivation and consumption are present), the EXTEM A-angle, CFR, and ACF sharply decline, indicating a hypocoagulable turnover due to excessive fibrinolysis and elevated D-dimers. ROC analyses revealed that A10-INTEM exhibits high specificity, while CFT-EXTEM and CFT-APTEM, A-angle-EXTEM, A10-EXTEM and A10-APTEM, and CFR-EXTEM demonstrate high sensitivity and specificity in predicting persistent thrombocytopenia. CFT-EXTEM demonstrates high specificity in managing both immediate and future platelet transfusions.

### 4.3. Neonates

ROTEM reveals that neonates born to women with PE show a hypocoagulable profile in the first hour of life compared with those of pregnant controls. INTEM and EXTEM reveal deficits in intrinsic and extrinsic factors, undetected by CCTs, while FIBTEM shows a fibrinogen deficit, confirmed by lower fibrinogen levels. Despite higher D-dimer levels, hyperfibrinolysis is not supported by APTEM/EXTEM. Hypocoagulability is not attributed to GA or a potential inflammatory response, as GA and CRP values are similar across groups. PE’s concomitant placental ischemia suppresses fetal megakaryocyte production due to reduced fetal bone-marrow perfusion, leading to neonatal thrombocytopenia. The latter is likewise supported by the observed decrease in white blood cell counts in our neonatal population born to mothers with PE, reflecting broader bone-marrow suppression. Likewise, PE’s placental ischemia impairs fetal liver function, leading to diminished production of coagulation factors [22,33,34,35,36]. Thus, neonatal hypocoagulability arises from low PLTs, a fibrinogen deficit, and coagulation factor deficits in neonates born to mothers with PE.

Neonates born to mothers with severe PE show a hypocoagulable profile analogous to that in the first analysis. Neonates born to mothers with non-severe PE exhibit slight hypocoagulability, with affected propagation observed within the first hour of life. High D-dimers in both groups suggest hyperfibrinolysis, which ROTEM does not confirm. Hypocoagulability in neonates born to mothers with PE is associated with PE’s severity. This is supported by the lower platelet counts noted in neonates whose mothers had severe PE. Neonates born to mothers with early-onset PE display hypocoagulability similar to that of neonates born to mothers with severe PE. In contrast, neonates born to mothers with late-onset PE are hypercoagulable, with affected amplification and propagation phases. Hyperfibrinolysis in both subgroups remains uncertain. GA may influence the results in the onset analysis; there is a de facto inherent imbalance since the definition of PE onset is based on GA. Neonates born to women with early-onset PE were, per se, of smaller GA and, thus, of lower birthweight, and those pregnancies were complicated by FGR. Therefore, FGR may also have an impact on these neonates’ hemostatic profiles. ROTEM reveals weaker and slower-forming clots in FGR neonates, and standard coagulation assays demonstrate prolonged clotting times and lower clotting factors in this population [37,38].

Viscoelastic tests in neonatal populations have been applied across various clinical spectra [24,25,39,40]. However, they are not typically used for neonates born to women with PE. This study is the first to assess hemostasis in neonates born exclusively to women with PE using viscoelastic tests. Existing studies show that, contrary to Kenny et al. [1], neonates born to women with PE often have lower PLTs and higher thrombocytopenia rates [22,41,42,43,44,45,46] linked to placental ischemia, megakaryocyte series suppression, and impaired megacaryocyte formation [43,45]. Fetal endothelial dysfunction may play an additional role in neonatal thrombocytopenia due to activation and consumption [44,47]. Fibrinogen results are mixed, with reports of both decreased [35] and elevated levels [42]. D-dimer levels are generally lower [35,48,49] or unchanged [47]. Neonates born to women with PE show reduced activity of coagulation factors II, V, VII, and XI, higher prothrombin activity, and lower prothrombin antigen [2,42]. Roes et al. observe elevated PAI-1 levels and lower uPA levels in neonates born to women with PE, indicating decreased fibrinolysis and a potential predisposition to thromboembolic complications. Plasminogen Activator Inhibitor 2 (PAI-2) does not differ [49]. However, Zanardo et al. found no differences in PAI-1, plasminogen, and tissue Plasminogen Activator (tPA), reporting elevated Protein C and lower Factor X antigens [35]. Other studies report no differences regarding α2-macroglobulin, Antithrombin III, t-PA, urokinase-type Plasminogen Activator (u-PA), PAI-1, and PAI-2 [2,47].

### 4.4. Limitations and Strengths

This study has some limitations. First, it is an observational study. Second, it is a one-time point study; we measured ROTEM parameters in mothers and neonates only once. The study lacks long-term follow-up, which can limit the generalizability of the findings. The number of mothers included in the severity and onset subgroup analyses was relatively small, resulting in inconclusive maternal findings for the above analyses. The fact that our inclusion criteria were very strict (excluding women with antiphospholipid syndrome, diabetes, etc.) led to the exclusion of many severe cases of PE; thus, we did not note the excessive hypercoagulability which other authors have described. Nevertheless, this limitation may be one of our strengths; the exclusion of populations with other comorbidities led to a clearer understanding of the contribution of PE per se to maternal and neonatal hemostatic alterations. Another major strength of our study is strict compliance with the protocol. To the best of our knowledge, this is one of the few studies assessing women with PE using ROTEM, and the first evaluating the hemostatic profile of neonates born exclusively to women with PE. Finally, a major strength of our study is that neonatal samples were obtained within the first hour of life, using a 21-G needle, before vitamin K administration, thereby enhancing the validity of the observed coagulation profiles. Neonatal arterial blood is an ideal choice; it requires no venipuncture tourniquet for collection, ensuring unobstructed blood flow and thus limiting hemostasis activation. Other studies have used cord blood, which can influence the reliability of hemostatic testing due to venous stasis, altering coagulation parameters, even if the sample is drawn shortly after cord clamping.

### 4.5. Future Implications

Our findings suggest that ROTEM in PE shows a pronounced hypercoagulable profile in mothers and a hypocoagulable profile in neonates, which correlates with disease severity. Viscoelastic testing might be incorporated into management regimens, enabling individualized hemostatic assessment at the bedside. ROTEM-guided transfusion algorithms, similar to those used in obstetric hemorrhage, can refine component therapy by targeting fibrinogen or platelet support based on FIBTEM or clot firmness metrics, reducing unnecessary blood product use and potentially improving outcomes. Serial ROTEM monitoring may also be used to track the evolution from compensated hypercoagulation to consumptive coagulopathy (as in HELLP), providing early warnings of imminent coagulopathy and directing delivery or escalation of therapy. More broadly, point-of-care ROTEM may aid in the stratification of maternal thrombotic vs. hemorrhagic risk and, indirectly, neonatal risk by indicating placental microvascular dysfunction. In summary, although larger studies are needed, these findings suggest that integrating ROTEM into preeclampsia care could enable individualized, goal-directed management, potentially reducing unnecessary transfusions and iatrogenic complications while balancing the dual risks of bleeding and thrombosis.

Thus, we think that future research should focus on the following areas:

- Large prospective studies should develop ROTEM reference patterns for PE and specify quantitative thresholds for predicting severe outcomes (e.g., progression to HELLP). This could confirm specific ROTEM parameters as predictive factors and improve maternal risk assessment.
- Trials are required to evaluate ROTEM-guided care methods in PE. Randomized trials could compare conventional care to ROTEM to administer tailored transfusions or to adjust delivery timing. Such trials would establish whether ROTEM-guided, tailored therapy improves maternal and newborn outcomes (by averting catastrophic bleeding or thrombosis) compared with standard management.

## 5. Conclusions

Although our study’s limitations include the sample size and lack of follow-up, our findings suggest that ROTEM provides vital insights into coagulation differences due to its dynamic nature. More studies with a larger sample and long-term follow-up need to focus on whether this may improve and guide clinical management of PE-related hemostatic issues in mothers and neonates.

While hypercoagulability is expected in women with PE, CCTs do not reveal its full extent; thus, ROTEM should be used alongside CCTs for hemostasis monitoring, transfusion management, and detection of hyperfibrinolysis. Viscoelastic tests typically show hypercoagulability until PLTs drop < 100 × 10^9^/L.

Hypocoagulability in neonates born to mothers with PE is unlikely to be due to prematurity, as no differences were observed between groups, and the calculated *p*-values were adjusted for gestational age. It may result from low PLTs, fibrinogen deficits, and coagulation factor deficiencies. PE’s concomitant placental ischemia could suppress fetal megakaryocytes production and impair fetal liver function, leading to diminished production of coagulation factors.

## Figures and Tables

**Table 1:** Baseline characteristics of pregnant women and their neonates (Preeclampsia group and controls). Summary measures are expressed as “Mean ± SD—Standard Deviation” or “Median (IQR—Interquartile Range)” for numerical variables and as Numbers with their respective percentages (%) for categorical variables. *p*-values in bold indicate statistical significance at the level α = 0.05.

Pregnant Women	PE ^1^ Group (N = 31)	Pregnant Controls (N = 45)	*p*-Value	Neonates	Neonates Born to Mothers with PE (N = 34)	Neonates Born to Pregnant Controls (N = 47)	*p*-Value
Age (years)	34 ± 5.9	33 ± 5.7	0.45	GA ^6^ (weeks)	33 (30–36)	36 (31.5–38)	0.07
Age above 40	4/31 (12.9%)	5/44 (11.4%)	1.00	Gender (male)	17/34 (50%)	26/47 (55.3%)	0.64
Race	All Caucasian	All Caucasian	-	Preterm	28/34 (82.3%)	32/47 (68.1%)	0.15
BMI ^2^ before (kg/m^2^)	26.3 (23.5–28.4)	23.3 (21.2–27.7)	0.06	Extremely preterm	4/28 (14.2%)	4/32 (12.5%)	1.00
BMI at labor (kg/m^2^)	29.6 (28.8–31.2)	28.3 (25.3–31.6)	0.12	Very preterm	8/28 (28.6%)	8/32 (25%)	0.76
BMI difference (kg/m^2^)	3.6 (1.7–5)	3.4 (2.5–5.5)	0.57	Moderate preterm	8/28 (28.6%)	8/32 (25%)	0.76
Smoking before pregnancy	7/31 (22.6%)	15/45 (33.3%)	0.31	Late preterm	8/28 (28.6%)	12/32 (37.5%)	0.46
Smoking during pregnancy	2/31 (6.5%)	9/45 (20%)	0.18	Birthweight (grams)	2035 (1475–2752.5)	2450 (1435–3140)	0.27
**Obstetric History**				Below 1000 g	7/34 (20.6%)	5/47 (10.6%)	0.21
Gravidity	2 (1–3)	2 (2–3)	0.06	Below 2500 g	22/34 (64.7%)	25/47 (53.2%)	0.30
Parity	1 (1–2)	2 (1–2)	**0.049**	Birthweight percentile	46.8 ± 30.7	45.3 ± 28	0.82
PE in previous pregnancies	3/31 (9.7%)	3/45 (6.7%)	0.68	Length (cm)	44.4 (36–49)	47 (40.5–50)	0.20
Pregnancy losses	0 (0–0.5)	1 (0–1)	**0.03**	Length percentile	57 (26.8–86.3)	56 (24.8–86.4)	0.79
**Drugs during Pregnancy**				Head circumference (cm)	31 (28.3–33.4)	32.5 (29.2–34.5)	0.39
Aspirin	21/31 (67,7%)	24/45 (53.3%)	0.21	Circumference percentile	54.5 (42.8–87.3)	68.9 (42.5–83.5)	0.86
LMWH ^3^	12/31 (38.7%)	16/45 (35.6%)	0.78	SGA ^7^	4/34 (11.8%)	5/47 (10.6%)	1.00
Progesterone	6/31 (19.4%)	11/45 (24.4%)	0.60	IUGR ^8^	8/34 (23.5%)	10/47 (21.3%)	0.81
Thyroxine	14/31 (45.2%)	17/45 (37.8%)	0.52	**Pregnancy Characteristics**			
Steroids	21/31 (67.7%)	28/45 (62.2%)	0.62	Twins	6/34 (17.7%)	4/47 (8.5%)	0.31
**Comorbidities and Major Complications**				ART ^9^	12/34 (35.3%)	13/47 (27.7%)	0.46
Hypothyroidism	14/31 (45.2%)	17/45 (37.8%)	0.52	Mean uterine artery’s PI ^10^	0.9 (0.8–1.5)	0.7 (0.6–0.9)	**0.01**
Hypercholesterolemia	1/31 (3.2%)	4/45 (8.9%)	0.64	Umbilical artery’s PI	1 ± 0.2	1 ± 0.1	0.77
Persistent thrombocytopenia after delivery	3/31 (9.7%)	0/45 (0%)	0.06	**Delivery Characteristics**			
Platelet transfusion	5/31 (16.1%)	0/45 (0%)	**0.009**	Delivery Type (CS ^11^)	33/34 (97.1%)	42/47 (89.4%)	0.39
ICU ^4^ admission	1/31 (3.2%)	0/45 (0%)	0.41	Placenta’s weight (grams)	385 (302–487.5)	450 (390–500)	0.10
Death	0/31 (0%)	0/45 (0%)	-	Umbilical cord’s clamp (seconds)	60 (42.5–60)	60 (40–60)	0.85
PE Type				Apgar score at 1 min	8 (7–8)	8 (7–8)	0.50
Early-onset PE	19/31 (61.3%)	NA	-	Apgar score at 5 min	8 (8–9)	9 (8–9)	**0.03**
Late-onset PE	12/31 (38.7%)	NA	-	**Neonatal Major Complications**			
Severe PE	11/31 (35.5%)	NA	-	NICU ^12^ admission	24/34 (70.6%)	24/47 (51.1%)	0.08
HELLP ^5^ syndrome	2/31 (6.5%)	NA	-	Respiratory assistance	21/34 (61.8%)	26/47 (55.3%)	0.56
				Infection	11/34 (32.4%)	9/47 (19.2%)	0.17
				Sepsis	4/34 (11.8%)	2/47 (4.3%)	0.23
				RDS ^13^	13/34 (38.2%)	14/47 (29.8%)	0.43
				BPD ^14^	5/34 (14.7%)	2/47 (4.3%)	0.12
				IVH ^15^	2/34 (5.9%)	2/47 (4.3%)	1.00
				NEC ^16^	2/34 (5.9%)	1/47 (2.1%)	0.57
				Thrombocytopenia	3/34 (8.8%)	1/47 (2.1%)	0.30
				Transfusion (blood/platelet/plasma)	7/34 (20.6%)	11/47 (23.4%)	0.76
				Death	1/34 (2.9%)	0/47 (0%)	0.42

^1^ PE: Preeclampsia; ^2^ BMI: Body Mass Index; ^3^ LMWH: Low-Molecular-Weight Heparin; ^4^ ICU: Intensive Care Unit; ^5^ Hemolysis, Elevated Liver enzymes, Low Platelets syndrome; ^6^ GA: Gestational Age at birth; ^7^ SGA: Small for Gestational Age; ^8^ IUGR: Intrauterine Growth Restriction; ^9^ ART: Assisted Reproductive Technology; ^10^ PI: Pulsatility Index; ^11^ CS: Caesarian Section; ^12^ NICU: Neonatal Intensive Care Unit; ^13^ RDS: Respiratory Distress Syndrome; ^14^ BPD: Bronchopulmonary Dysplasia; ^15^ IVH: Intraventricular Hemorrhage; ^16^ NEC: Necrotizing Enterocolitis. Extremely preterm: born 22 GW to 27^+6^ GW; very preterm: born 28 GW to 31^+6^ GW; moderate preterm: born 32 GW to 33^+6^ GW; late preterm: born 34 GW to 36^+6^ GW.

**Table 2:** Maternal and neonatal results (Preeclampsia group and controls). Neonatal *p*-values are adjusted for the following covariates: Gestational Age and Apgar score at 5 min. Summary measures are expressed as “Mean ± SD—Standard Deviation” or “Median (IQR—Interquartile Range)” for numerical variables and as numbers with their respective percentages (%) for categorical variables. *p*-values in bold indicate statistical significance at the level α = 0.05.

Pregnant Women		PE ^1^ Group (N = 31)	Pregnant Controls (N = 45)	*p*-Value	Neonates		Neonates Born to Mothers with PE (N = 34)	Neonates Born to Pregnant Controls (N = 47)	**Adjusted *p*-Value (Adjusted for GA ^20^ and Apgar Score at 5 min)**
Platelet count × 10^9^/L		167 (138.5–201.5)	234 (197–271)	**<0.001**	Platelet count × 10/L		230 (168–257)	282 (226.5–322.5)	**0.002**
MPV ^2^ (fL)		9.8 (8.4–10.8)	10.2 (9.4–10.7)	0.61	MPV (fL)		7.4 (6.9–8)	9.1 (7.4–9.8)	**0.01**
Platelet count < 100 × 10^9^/L		3/31 (9.7%)	0/45 (0%)	0.06	Platelet count < 100 × 10^9^/L		0/33 (0%)	0/47 (0%)	-
Platelet count < 150 × 10^9^/L		12/31 (38.7%)	1/45 (2.2%)	**<0.001**	Platelet count < 150 × 10^9^/L		5/33 (15.2%)	1/47 (2.1%)	0.08
Antithrombin III (%)		92.8 ± 14.8	97.6 ± 9.9	0.13	-		-	-	-
Protein C activity (%)		101 (86.5–115.5)	103 (97–111)	0.29	-		-	-	-
Free Protein S Ag (%)		28.7 ± 4.9	39.5 ± 6.3	**<0.001**	-		-	-	-
**CCTs ^3^**	PT ^8^ (seconds)	12.5 (12.1–13.1)	12.8 (12.3–13.4)	0.28	**CCTs**	PT (seconds)	14.3 (13.4–16.5)	14.6 (13.4–15.6)	0.87
	APTT ^9^ (seconds)	29.1 (26.7–30.3)	28.5 (27.4–29.5)	0.44		APTT (seconds)	42 (37.3–52.9)	42.5 (38.2–48.6)	0.70
	INR ^10^	0.92 (0.91–0.96)	0.95 (0.92–0.99)	0.11		INR	1.06 (0.99–1.19)	1.08 (0.98–1.14)	0.76
	Fibrinogen (mg/dL)	514.6 ± 110.8	456.5 ± 108.8	**0.03**		Fibrinogen (mg/dL)	208.5 (161.5–276)	214 (178.3–280.5)	0.68
	D-Dimers (ng/mL)	1980 (1610–2600)	1122.5 (880–1565)	**<0.001**		D-Dimers (ng/mL)	2003.5 (907.5–2702.5)	870 (571–1200)	**<0.001**
**INTEM ^4^**	CT ^11^ (seconds)	163.4 ± 26.3	169.9 ± 29	0.32	**INTEM**	CT (seconds)	217 (197.3–250.8)	214 (189.5–247)	0.66
	CFT ^12^ (seconds)	60 (48.5–65.5)	64 (54–73)	0.19		CFT (seconds)	98 (88–128.8)	77 (64–119.5)	**0.005**
	MCF ^13^ (mm)	73 (69–74.5)	70 (66–72)	0.053		MCF (mm)	50.4 ± 6.2	55.7 ± 8.4	**0.003**
	A-angle (° degrees)	79 (77–80)	77 (76–79)	0.08		A-angle (° degrees)	71.5 (67.3–74)	75 (69–77)	**0.01**
	A10 ^14^ (mm)	65 (60.5–68)	62 (57–65)	0.14		A10 (mm)	44.9 ± 6.7	50.7 ± 9.5	**0.003**
	A30 ^14^ (mm)	71 (68.5–74.5)	69 (66–72)	0.10		A30 (mm)	49.9 ± 6.1	55 ± 8.2	**0.003**
	MCE ^15^ (dynes/cm^2^)	255 (224–294)	236 (194–258)	0.08		MCE (dynes/cm^2^)	100 (90.5–114.5)	131 (95.5–159)	**0.003**
	LI60 ^16^ (%)	97 (97–100)	97 (95–98.25)	0.06		LI60 (%)	91.9 ± 3.3	92.2 ± 3.5	0.65
	ML ^17^ (%)	4.3 ± 3.2	6.2 ± 3.9	**0.03**		ML (%)	12 (9.3–16)	11 (8–15.5)	0.44
	CFR ^18^ (° degrees)	80 (79–81.5)	79 (77–80)	**0.03**		CFR (° degrees)	74 (70–76)	76 (70.5–78.5)	0.06
	ACF ^19^ (mm)	69 (66.3–71)	65 (61–69)	**0.03**		ACF (mm)	44.3 ± 5.9	49.2 ± 8.9	**0.003**
**EXTEM ^5^**	CT (seconds)	58 (53.5–62)	58 (52–62)	0.79	**EXTEM**	CT (seconds)	58 (52.3–65)	56 (51–67)	0.53
	CFT (seconds)	61 (49–78.5)	68 (56–79)	0.39		CFT (seconds)	121 (97–155)	94 (75.5–146.5)	**0.02**
	MCF (mm)	69 (66.5–75)	71 (67–73)	0.82		MCF (mm)	51 (44–54.8)	56 (47.5–61)	**0.006**
	A-angle (° degrees)	78.5 (75–80)	77 (75–79)	0.19		A-angle (° degrees)	67 (64.3–71)	71 (65–74.5)	**0.04**
	A10 (mm)	63 (59.5–68.5)	62 (58–66)	0.55		A10 (mm)	44.5 (37.3–48)	51 (40–55.5)	**0.009**
	A30 (mm)	69 (66.5–74)	70 (66–72)	0.93		A30 (mm)	50.5 (44–54)	55 (47–60)	**0.01**
	MCE (dynes/cm^2^)	230.1 ± 76.1	237.9 ± 53.3	0.60		MCE (dynes/cm^2^)	103 (77.8–120.5)	126 (90.5–154)	**0.007**
	LI60 (%)	95 (89–98)	96 (92.8–98)	0.32		LI60 (%)	90 (87–92)	92 (89–94)	**0.004**
	ML (%)	9 (4.5–14.5)	8 (5–13)	0.83		ML (%)	17.5 (12.3–26)	13 (9.5–19.5)	0.06
	CFR (° degrees)	79.5 (76–81)	78 (76–80)	0.26		CFR (° degrees)	71 (68–74)	74 (67–76.5)	0.07
	ACF (mm)	62 (57.5–66)	64 (59–67)	0.34		ACF (mm)	40 (34.3–45)	45 (41–53)	**0.001**
**FIBTEM ^6^**	CT (seconds)	52 (49–57.5)	57 (53–67)	**0.02**	**FIBTEM**	CT (seconds)	58.5 (51.3–73.8)	60 (50.5–71)	0.96
	CFT (seconds)	-	-	-		CFT (seconds)	-	-	-
	MCF (mm)	27.7 ± 7.5	24.5 ± 6.3	0.051		MCF (mm)	12 (9–14.8)	16 (11.5–18.5)	**0.009**
	A-angle (° degrees)	77 (74–79)	75 (71.8–77.3)	**0.04**		A-angle (° degrees)	65.8 ± 6.2	69.2 ± 6.9	0.19
	A10 (mm)	25.3 ± 7.7	22.3 ± 6.2	0.07		A10 (mm)	11 (9–13)	14 (9.5–16.5)	**0.009**
	A30 (mm)	27.5 ± 7.6	24.4 ± 6.3	0.06		A30 (mm)	12 (9.3–15)	16 (11.5–19)	**0.009**
	MCE (dynes/cm^2^)	39.8 ± 15.6	33.4 ± 11.8	**0.04**		MCE (dynes/cm^2^)	13.5 (10–16)	19 (12.5–23)	**0.005**
	LI60 (%)	100 (99–100)	100 (99–100)	0.45		LI60 (%)	97 (91.3–100)	96.5 (92.3–100)	0.87
	ML (%)	1 (0–2)	0 (0–2)	0.17		ML (%)	8 (2–13.8)	7 (1–12.5)	0.62
	CFR (° degrees)	78.5 (75–80.8)	76 (73–78)	**0.02**		CFR (° degrees)	67.9 ± 5.6	70.7 ± 5.7	0.17
	ACF (mm)	27.5 ± 7.1	24.6 ± 5.8	**0.04**		ACF (mm)	11 (9–12)	14 (10–17.5)	**0.01**
**APTEM ^7^**	CT (seconds)	55 (51–59.5)	59 (54–67)	**0.03**	**APTEM**	CT (seconds)	58 (50–66.8)	57 (47.5–70)	0.92
	CFT (seconds)	69 (52.5–117)	75 (65–86)	0.26		CFT (seconds)	133 (105–164)	97 (79.5–143)	**0.007**
	MCF (mm)	72 (66.5–74)	68 (65–71)	0.06		MCF (mm)	48 (41.3–53)	54 (46–58)	**0.002**
	A-angle (° degrees)	78 (74.5–80)	75 (73–77)	**0.003**		A-angle (° degrees)	67 (62.5–71)	71 (65–74)	0.08
	A10 (mm)	62 (56–67)	58 (55–62)	0.08		A10 (mm)	39.8 ± 9.3	47.1 ± 9.5	**0.001**
	A30 (mm)	71 (65–74)	67 (64–70)	0.06		A30 (mm)	46.2 ± 9.5	52.8 ± 8	**0.001**
	MCE (dynes/cm^2^)	236.6 ± 74.5	214.5 ± 54.3	0.14		MCE (dynes/cm^2^)	92 (69.3–113.5)	119 (85.5–138.5)	**0.002**
	LI60 (%)	98 (97–99.3)	98 (96.8–99)	0.35		LI60 (%)	90 (82.3–93)	93 (89.3–94.8)	**0.008**
	ML (%)	4 (3–8)	6 (3–9)	0.31		ML (%)	19.5 (13–27)	14 (9–19)	**0.005**
	CFR (° degrees)	79 (76.5–81)	76 (74–78)	**0.001**		CFR (° degrees)	71 (67–74)	73 (68–76)	0.22
	ACF (mm)	67 (61–71)	65 (59–68)	0.13		ACF (mm)	37 (32–43.8)	45 (39.5–51.5)	**<0.001**

^1^ PE: Preeclampsia; ^2^ MPV: Mean Platelet Volume; ^3^ CCTs: Conventional Coagulation Tests; ^4^ INTEM: Intrinsic Thromboelastometry; ^5^ EXTEM: Extrinsic Thromboelastometry; ^6^ FIBTEM: Fibrinogen Thromboelastometry; ^7^ APTEM: Aprotinin Thromboelastometry; ^8^ PT: Prothrombin Time; ^9^ APTT: Activated Partial Thromboplastin Time; ^10^ INR: International Normalized Ratio; ^11^ CT: Clotting Time; ^12^ CFT: Clot Formation Time; ^13^ MCF: Maximum Clot Firmness; ^14^ A10, 30: Amplitude at 10, 30 min after Clotting Time (post CT); ^15^ MCE: Maximum Clot Elasticity; ^16^ LI60: Lysis Index at 60 min after Clotting Time (post CT); ^17^ ML: Maximum Lysis; ^18^ CFR: Clot Formation Rate; ^19^ ACF: Actual Clot Firmness; ^20^ GA: Gestational Age.

**Table 3:** Neonatal results regarding preeclampsia severity (non-severe Preeclampsia subgroup, severe Preeclampsia subgroup, and controls). Summary measures are expressed as “Mean ± SD—Standard Deviation” or “Median (IQR—Interquartile Range)” for numerical variables and as Numbers with their respective percentages (%) for categorical variables. The *p*-values are adjusted for Gestational Age. *p*-values in bold indicate statistical significance at the level α = 0.05.

Neonates		Neonates Born to Mothers with Non-Severe PE ^1^ (N = 13)	Neonates Born to Mothers with Severe PE (N = 13)	Neonates Born to Pregnant Controls (N = 21)	Adjusted *p*-Value (Adjusted for Gestational Age)			
					Across Subgroups	Non-Severe PE vs. Severe PE	Non-Severe PE vs. Controls	Severe PE vs. Controls
Platelet count × 10^9^/L		229 ± 49.3	198.8 ± 60.9	264 ± 53.3	**0.004**	0.65	0.14	**0.004**
MPV ^2^ (fL)		7.7 (7.1–8)	7.8 (6.7–9.2)	9 (7.4–9.8)	0.15	-	-	-
Platelet count < 100 × 10^9^/L		0/13 (0%)	0/13 (0%)	0/21 (0%)	-	-	-	-
Platelet count < 150 × 10^9^/L		1/13 (7.7%)	3/13 (23.1%)	1/21 (4.8%)	0.36	-	-	-
**CCTs ^3^**	PT ^8^ (seconds)	15.6 (13.7–16.4)	14.1 (13.4–14.8)	15.8 (15–17.2)	0.16	-	-	-
	APTT ^9^ (seconds)	44.3 (38.8–47.4)	42 (37.1–53.2)	46.4 (41.8–55.2)	0.50	-	-	-
	INR ^10^	1.12 (1–1.18)	1.04 (0.99–1.1)	1.15 (1.09–1.26)	0.17	-	-	-
	Fibrinogen (mg/dL)	193 (159–261)	185 (160–253)	210 (161.5–293.8)	0.87	-	-	-
	D-Dimers (ng/mL)	1550 (1020–2210)	2340 (1900–2560)	950 (680–1650)	**0.003**	0.68	**0.03**	**0.003**
**INTEM ^4^**	CT ^11^ (seconds)	218 (189–247)	206 (196–255)	203 (188–247)	0.73	-	-	-
	CFT ^12^ (seconds)	88 (77–106)	110 (96–131)	70 (62–87)	**0.002**	0.22	0.12	**0.001**
	MCF ^13^ (mm)	51.9 ± 5.5	48.5 ± 4.9	56.7 ± 8.2	**0.005**	0.72	0.14	**0.004**
	A-angle (° degrees)	73 (70–74)	69 (65–72)	76 (72–77)	**0.008**	0.38	0.16	**0.005**
	A10 ^14^ (mm)	47 (44–51)	43 (41–45)	54 (48–59)	**<0.001**	0.15	0.09	**<0.001**
	A30 ^14^ (mm)	51.5 ± 5.3	47.9 ± 4.9	56.1 ± 8.3	**0.005**	0.57	0.18	**0.004**
	MCE ^15^ (dynes/cm^2^)	110.9 ± 26.4	94.5 ± 17.2	137.6 ± 39.7	**0.001**	0.62	0.06	**0.001**
	LI60 ^16^ (%)	92 (90–93)	93 (92–94)	93 (92–96)	0.40	-	-	-
	ML ^17^ (%)	11.5 ± 4.1	11.9 ± 4.4	10 ± 4	0.38	-	-	-
	CFR ^18^ (° degrees)	75 (72–77)	71 (67–74)	77 (74–79)	**0.02**	0.45	0.22	**0.01**
	ACF ^19^ (mm)	45.8 ± 4.9	42.9 ± 4.5	51.2 ± 8.4	**0.003**	0.76	0.10	**0.003**
**EXTEM ^5^**	CT (seconds)	57 (50–65)	58 (56–71)	52 (49–64)	0.50	-	-	-
	CFT (seconds)	119 (97–127)	127 (101.8–175.5)	91 (77–114)	**0.009**	0.47	0.13	**0.006**
	MCF (mm)	52 (49–55)	49 (38–51)	57 (48–61)	**0.005**	0.24	0.20	**0.003**
	A-angle (° degrees)	68 (66–71)	67 (64–71)	72 (69–74)	0.07	-	-	-
	A10 (mm)	45 (44–50)	42 (34–45)	52 (42–55)	**0.01**	0.42	0.17	**0.006**
	A30 (mm)	52 (48–54)	48 (38–51)	57 (47–60)	**0.006**	0.22	0.25	**0.003**
	MCE (dynes/cm^2^)	109.9 ± 26	85.7 ± 29.8	129.4 ± 39.3	**0.002**	0.31	0.18	**0.001**
	LI60 (%)	91 (90–92)	89 (82–90)	94 (91–95)	**0.008**	0.21	0.32	**0.004**
	ML (%)	17 (11–23)	16 (14–26)	11 (6–15)	0.06	-	-	-
	CFR (° degrees)	71 (69–75)	71 (68–73)	74 (72–76)	0.16	-	-	-
	ACF (mm)	43 (37–47)	39 (30–43)	50 (44–53)	**<0.001**	0.22	0.07	**<0.001**
**FIBTEM ^6^**	CT (seconds)	51 (49–59)	59 (53–63)	56 (45–72)	0.53	-	-	-
	CFT (seconds)	-	-	-	-	-	-	-
	MCF (mm)	13 (11–14)	10 (9–14)	18 (14–19)	**0.003**	0.48	0.06	**0.002**
	A-angle (° degrees)	65 (62–68.5)	70 (70–70)	70.5 (65.3–74)	0.49	-	-	-
	A10 (mm)	12 (11–14)	9 (8–11)	15 (13–18)	**<0.001**	0.20	0.06	**<0.001**
	A30 (mm)	13 (12–15)	10 (9–14)	18 (14–19)	**0.002**	0.47	**0.048**	**0.002**
	MCE (dynes/cm^2^)	15 (13–16)	11 (10–14)	21 (16–24)	**<0.001**	0.22	0.06	**<0.001**
	LI60 (%)	96 (91–100)	96 (90–100)	100 (95–100)	0.18	-	-	-
	ML (%)	9 (3–16)	12 (2–14)	3 (0–8)	0.15	-	-	-
	CFR (° degrees)	68 (65–70)	73 (73–73)	71.5 (66.5–75)	0.39	-	-	-
	ACF (mm)	12 ± 3.2	10 ± 2.4	16.3 ± 7.4	**0.05**	1.00	0.10	**0.006**
**APTEM ^7^**	CT (seconds)	52 (49–66)	59 (55–67)	54 (48–70)	0.65	-	-	-
	CFT (seconds)	135 (105–157)	139 (121.5–164.3)	95 (79–143)	**0.02**	0.88	0.08	**0.02**
	MCF (mm)	49 (45–53)	46 (41–50)	56 (48–61)	**0.006**	0.88	**0.03**	**0.008**
	A-angle (° degrees)	68 (65–72)	65 (62–67)	71 (66–74)	0.11	-	-	-
	A10 (mm)	40.3 ± 7.4	38.4 ± 10.7	48.2 ± 8.7	**0.005**	1.00	**0.04**	**0.01**
	A30 (mm)	49 (44–53)	46 (40–50)	56 (47–61)	**0.005**	0.91	**0.02**	**0.007**
	MCE (dynes/cm^2^)	92.9 ± 23.7	85.9 ± 29.3	126.6 ± 37.3	**0.001**	1.00	**0.01**	**0.003**
	LI60 (%)	90.7 ± 5.7	86.3 ± 8.1	93.6 ± 4.7	**0.004**	0.12	0.72	**0.003**
	ML (%)	18 (8–22)	21 (14–29)	11 (6–16)	**0.008**	0.20	0.34	**0.004**
	CFR (° degrees)	72.5 (69–74.5)	68 (66–73)	73 (69–76)	0.30	-	-	-
	ACF (mm)	40 (36–46)	36 (32–42)	50 (41–56)	**<0.001**	0.26	**0.009**	**<0.001**

^1^ PE: Preeclampsia; ^2^ MPV: Mean Platelet Volume; ^3^ CCTs: Conventional Coagulation Tests; ^4^ INTEM: Intrinsic Thromboelastometry; ^5^ EXTEM: Extrinsic Thromboelastometry; ^6^ FIBTEM: Fibrinogen Thromboelastometry; ^7^ APTEM: Aprotinin Thromboelastometry; ^8^ PT: Prothrombin Time; ^9^ APTT: Activated Partial Thromboplastin Time; ^10^ INR: International Normalized Ratio; ^11^ CT: Clotting Time; ^12^ CFT: Clot Formation Time; ^13^ MCF: Maximum Clot Firmness; ^14^ A10, 30: Amplitude at 10, 30 min after Clotting Time (post CT); ^15^ MCE: Maximum Clot Elasticity; ^16^ LI60: Lysis Index at 60 min after Clotting Time (post CT); ^17^ ML: Maximum Lysis; ^18^ CFR: Clot Formation Rate; ^19^ ACF: Actual Clot Firmness.

## Data Availability

The data presented in this study are available on request from the corresponding author due to privacy and ethical restrictions.

## Supplementary Materials

The following supporting information can be downloaded at: https://www.mdpi.com/article/10.3390/diagnostics15172156/s1, Table S1. This table shows the Rotational Thromboelastometry (ROTEM) assays, their relevant details (how clot formation is induced in each assay), the ROTEM parameters measured in this study, their respective measurement units, and what each parameter reflects. Table S2. Maternal and neonatal blood count and biochemical tests’ results (Preeclampsia Group and Controls). Summary measures are expressed as “Mean ± SD—Standard Deviation” or “Median (IQR—Interquartile Range)” for numerical variables and as Numbers with their respective percentages (%) for categorical variables. *p*-values in bold indicate statistical significance at the level α = 0.05. Table S3. Baseline characteristics of pregnant women regarding Preeclampsia’s severity (Non-Severe Preeclampsia subgroup, Severe Preeclampsia subgroup and Controls). Summary measures are expressed as “Mean ± SD—Standard Deviation” or “Median (IQR—Interquartile Range)” for numerical variables and as Numbers with their respective percentages (%) for categorical variables. *p*-values in bold indicate statistical significance at the level α = 0.05. Table S4. Maternal blood count and maternal biochemical tests’ results regarding Preeclampsia’s severity (Non-Severe Preeclampsia subgroup, Severe Preeclampsia subgroup and Controls). Summary measures are expressed as “Mean ± SD—Standard Deviation” or “Median (IQR—Interquartile Range)” for numerical variables and as Numbers with their respective percentages (%) for categorical variables. *p*-values in bold indicate statistical significance at the level α = 0.05. Table S5. Maternal results regarding Preeclampsia’s severity (Non-Severe Preeclampsia subgroup, Severe Preeclampsia subgroup and Controls). Summary measures are expressed as “Mean ± SD—Standard Deviation” or “Median (IQR—Interquartile Range)” for numerical variables and as Numbers with their respective percentages (%) for categorical variables. *p*-values in bold indicate statistical significance at the level α = 0.05. Table S6. Baseline characteristics of neonates regarding Preeclampsia’s severity (Non-Severe Preeclampsia subgroup, Severe Preeclampsia subgroup and Controls). Summary measures are expressed as “Mean ± SD—Standard Deviation” or “Median (IQR—Interquartile Range)” for numerical variables and as Numbers with their respective percentages (%) for categorical variables. *p*-values in bold indicate statistical significance at the level α = 0.05. Table S7. Neonatal blood count and neonatal biochemical tests’ results regarding Preeclampsia’s severity (Non-Severe Preeclampsia subgroup, Severe Preeclampsia subgroup and Controls). Summary measures are expressed as “Mean ± SD—Standard Deviation” or “Median (IQR—Interquartile Range)” for numerical variables and as Numbers with their respective percentages (%) for categorical variables. *p*-values in bold indicate statistical significance at the level α = 0.05. Table S8. Baseline characteristics of pregnant women when divided into Early- and Late-Onset Preeclampsia subgroups (Early-Onset Preeclampsia subgroup, Late-Onset Preeclampsia subgroup and Controls). Summary measures are expressed as “Mean ± SD—Standard Deviation” or “Median (IQR—Interquartile Range)” for numerical variables and as Numbers with their respective percentages (%) for categorical variables. *p*-values in bold indicate statistical significance at the level α = 0.05. Table S9. Maternal blood count and maternal biochemical tests’ results when pregnant women are divided into Early- and Late-Onset Preeclampsia subgroups (Early-Onset Preeclampsia subgroup, Late-Onset Preeclampsia subgroup and Controls). Summary measures are expressed as “Mean ± SD—Standard Deviation” or “Median (IQR—Interquartile Range)” for numerical variables and as Numbers with their respective percentages (%) for categorical variables. *p*-values in bold indicate statistical significance at the level α = 0.05. Table S10. Maternal results when pregnant women are divided into Early- and Late-Onset Preeclampsia subgroups (Early-Onset Preeclampsia subgroup, Late-Onset Preeclampsia subgroup and Controls). Summary measures are expressed as “Mean ± SD—Standard Deviation” or “Median (IQR—Interquartile Range)” for numerical variables and as Numbers with their respective percentages (%) for categorical variables. *p*-values in bold indicate statistical significance at the level α = 0.05. Table S11. Baseline characteristics of neonates regarding Preeclampsia’s onset (Early-Onset Preeclampsia subgroup, Late-Onset Preeclampsia subgroup and Controls). Summary measures are expressed as “Mean ± SD—Standard Deviation” or “Median (IQR—Interquartile Range)” for numerical variables and as Numbers with their respective percentages (%) for categorical variables. *p*-values in bold indicate statistical significance at the level α = 0.05. Table S12. Neonatal blood count and neonatal biochemical tests’ results regarding Preeclampsia’s onset (Early-Onset Preeclampsia subgroup, Late-Onset Preeclampsia subgroup and Controls). Summary measures are expressed as “Mean ± SD—Standard Deviation” or “Median (IQR—Interquartile Range)” for numerical variables and as Numbers with their respective percentages (%) for categorical variables. *p*-values in bold indicate statistical significance at the level α = 0.05. Table S13. Neonatal results regarding Preeclampsia’s severity Preeclampsia’s onset (Early-Onset Preeclampsia subgroup, Late-Onset Preeclampsia subgroup and Controls). This table presents the unadjusted and adjusted *p*-values. Adjusted *p*-values are presented only for the parameters that differed statistically. The *p*-values are adjusted for the following covariates: Gestational Age, Birthweight, Length, Head Circumference, Apgar score at 1 and 5 min. *p*-values in bold indicate statistical significance at the level α = 0.05. Table S14. All Spearman correlations between Platelet Count, Fibrinogen, D-dimers and Rotational Thromboelastometry (ROTEM) parameters regarding pregnant women. This table shows the correlations that were moderate (0.40 ≤ |ρ| ≤ 0.59), strong (0.60 ≤ |ρ| ≤ 0.79) or very strong (0.80 ≤ |ρ|) and with a *p*-value ≤ 0.05. Table S15. All Spearman correlations between Platelet Count, Fibrinogen and Rotational Thromboelastometry (ROTEM) parameters regarding neonates. This table shows the correlations that were moderate (0.40 ≤ |ρ| ≤ 0.59), strong (0.60 ≤ |ρ| ≤ 0.79) or very strong (0.80 ≤ |ρ|) and with a *p*-value ≤ 0.05. Table S16. Regression models for strong and very strong correlations regarding pregnant women with Preeclampsia. The selection of the model was based on the statistical significance of the *p*-values of the intercept and the coefficients and the adjusted R^2^. For each correlation the selected model is highlighted in yellow. *p*-values in bold indicate statistical significance at the level α = 0.05. Table S17. All logistic regressions with *p*-value ≤ 0.05 regarding pregnant women with Preeclampsia and neonates born to pregnant women with Preeclampsia. Table S18. All variables for which a Receiver Operating Characteristic (ROC) curve was created based on the logistic regression’s results. *p*-values in bold indicate statistical significance at the level α = 0.05.

## Abbreviations

The following abbreviations are used in this manuscript:

A10, 20, 30	Amplitude at 10, 20, 30 min after Clotting Time
ACF	Actual Clot Firmness
ALT	Alanine Aminotransferase
APTEM	Aprotinin Rotational Thromboelastometry
APTT	Activated Partial Thromboplastin Time
AST	Aspartate Aminotransferase
AUC	Area Under Curve
CCTs	Conventional Coagulation Tests
CFR	Clot Formation Rate
CFT	Clot Formation Time
CI	Coagulation Index
CT	Clotting Time
EXTEM	Extrinsic Rotational Thromboelastometry
FIBTEM	Fibrinogen Rotational Thromboelastometry
GA	Gestational Age
GWs	Gestational Weeks
HELLP	Hemolysis, Elevated Liver enzymes, Low Platelets
HEPTEM	Heparinase Rotational Thromboelastometry
INR	International Normalized Ratio
INTEM	Intrinsic Rotational Thromboelastometry
K	Clot Kinetics
LDH	Lactate Dehydrogenase
LI60	Lysis Index at 60 min after Clotting Time
LY60	Lysis Index or Clot Lysis at 60 min after Maximum Amplitude
MA	Maximum Amplitude
MCE	Maximum Clot Elasticity
MCF	Maximum Clot Firmness
ML	Maximum Lysis
PAI-1, 2	Plasminogen Activator Inhibitor 1, 2
PE	Preeclampsia
PLT(s)	Platelet(s)
PT	Prothrombin Time
R	Reaction Time
ROC	Receiver Operating Characteristic
ROTEM	Rotational Thromboelastometry
TEG	Thromboelastography
tPA	tissue Plasminogen Activator
uPA	urokinase-type Plasminogen Activator
