# Supplementary material for: Rotational Thromboelastometry (ROTEM) Hemostasis Profile in Pregnant Women with Preeclampsia and Their Offspring: An Observational Study
Source: Diagnostics (Basel). 2025 Aug 26;15(17):2156. doi: 10.3390/diagnostics15172156 (PMC12428756; doi:10.3390/diagnostics15172156)
Supplement: Supplementary file 1 [file diagnostics-15-02156-s001.zip › diagnostics-3792729-supplementary.pdf]

SUPPORTING INFORMATION

SUPPLEMENTARY TABLES

Table S1. This table shows the Rotational Thromboelastometry (ROTEM) assays, their relevant details (how clot formation is induced in each assay), the ROTEM parameters measured in this study, their respective measurement units, and what each parameter reflects. 4

Table S2. Maternal and neonatal blood count and biochemical tests' results (Preeclampsia Group and Controls). Summary measures are expressed as "Mean ± SD – Standard Deviation" or "Median (IQR – Interquartile Range)" for numerical variables and as Numbers with their respective percentages (%) for categorical variables. P-values in bold indicate statistical significance at the level α=0.05.. 5

Table S3. Baseline characteristics of pregnant women regarding Preeclampsia's severity (Non-Severe Preeclampsia subgroup, Severe Preeclampsia subgroup and Controls). Summary measures are expressed as "Mean ± SD – Standard Deviation" or "Median (IQR – Interquartile Range)" for numerical variables and as Numbers with their respective percentages (%) for categorical variables. P-values in bold indicate statistical significance at the level α=0.05..... 6

Table S4. Maternal blood count and maternal biochemical tests' results regarding Preeclampsia's severity (Non-Severe Preeclampsia subgroup, Severe Preeclampsia subgroup and Controls). Summary measures are expressed as "Mean ± SD – Standard Deviation" or "Median (IQR – Interquartile Range)" for numerical variables and as Numbers with their respective percentages (%) for categorical variables. P-values in bold indicate statistical significance at the level α=0.05.. 7

Table S5. Maternal results regarding Preeclampsia's severity (Non-Severe Preeclampsia subgroup, Severe Preeclampsia subgroup and Controls). Summary measures are expressed as "Mean ± SD – Standard Deviation" or "Median (IQR – Interquartile Range)" for numerical variables and as Numbers with their respective percentages (%) for categorical variables. P-values in bold indicate statistical significance at the level α=0.05...................................... 8

Table S6. Baseline characteristics of neonates regarding Preeclampsia's severity (Non-Severe Preeclampsia subgroup, Severe Preeclampsia subgroup and Controls). Summary measures are expressed as "Mean ± SD – Standard Deviation" or "Median (IQR – Interquartile Range)" for numerical variables and as Numbers with their respective percentages (%) for categorical variables. P-values in bold indicate statistical significance at the level α=0.05. 10

Table S7. Neonatal blood count and neonatal biochemical tests' results regarding Preeclampsia's severity (Non-Severe Preeclampsia subgroup, Severe Preeclampsia subgroup and Controls). Summary measures are expressed as "Mean ± SD – Standard Deviation" or "Median (IQR – Interquartile Range)" for numerical variables and as Numbers with their respective percentages (%) for categorical variables. P-values in bold indicate statistical significance at the level α=0.05...12

Table S8. Baseline characteristics of pregnant women when divided into Early- and Late-Onset Preeclampsia subgroups (Early-Onset Preeclampsia subgroup, Late-Onset Preeclampsia subgroup and Controls). Summary measures are expressed as "Mean ± SD – Standard Deviation" or "Median (IQR – Interquartile Range)" for numerical variables and as Numbers with their respective percentages (%) for categorical variables. P-values in bold indicate statistical significance at the level α=0.05. 13

Table S9. Maternal blood count and maternal biochemical tests' results when pregnant women are divided into Early- and Late-Onset Preeclampsia subgroups (Early-Onset Preeclampsia subgroup, Late-Onset Preeclampsia subgroup and Controls). Summary measures are expressed as "Mean ± SD – Standard Deviation" or "Median (IQR – Interquartile Range)" for numerical variables and as Numbers with their respective percentages (%) for categorical variables. P-values in bold indicate statistical significance at the level α=0.05.....................................14

Table S10. Maternal results when pregnant women are divided into Early- and Late-Onset Preeclampsia subgroups (Early-Onset Preeclampsia subgroup, Late-Onset Preeclampsia subgroup and Controls). Summary measures are expressed as "Mean ± SD – Standard Deviation" or "Median (IQR – Interquartile Range)" for numerical variables and as Numbers with their respective percentages (%) for categorical variables. P-values in bold indicate statistical significance at the level α=0.05. 15

Table S11. Baseline characteristics of neonates regarding Preeclampsia's onset (Early-Onset Preeclampsia subgroup, Late-Onset Preeclampsia subgroup and Controls). Summary measures are expressed as "Mean ± SD – Standard Deviation" or "Median (IQR – Interquartile Range)" for numerical variables and as Numbers with their respective percentages (%) for categorical variables. P-values in bold indicate statistical significance at the level α=0.05. 17

Table S12. Neonatal blood count and neonatal biochemical tests' results regarding Preeclampsia's onset (Early-Onset Preeclampsia subgroup, Late-Onset Preeclampsia subgroup and Controls). Summary measures are expressed as "Mean ± SD – Standard Deviation" or "Median (IQR – Interquartile Range)" for numerical variables and as Numbers with their respective percentages (%) for categorical variables. P-values in bold indicate statistical significance at the level α=0.05...19

Table S13. Neonatal results regarding Preeclampsia's severity Preeclampsia's onset (Early-Onset Preeclampsia subgroup, Late-Onset Preeclampsia subgroup and Controls). This table presents the unadjusted and adjusted p-values. Adjusted p-values are presented only for the parameters that differed statistically. The p-values are adjusted for the following covariates: Gestational Age, Birthweight, Length, Head Circumference, Apgar score at 1 and 5 minutes. P-values in bold indicate statistical significance at the level α=0.05.....................................20

Table S14. All Spearman correlations between Platelet Count, Fibrinogen, D-dimers and Rotational Thromboelastometry (ROTEM) parameters regarding pregnant women. This table shows the correlations that were moderate (0.40 ≤ |ρ| ≤ 0.59), strong (0.60 ≤ |ρ| ≤ 0.79) or very strong (0.80 ≤ |ρ|) and with a P-value ≤0.05. 22

Table S15. All Spearman correlations between Platelet Count, Fibrinogen and Rotational Thromboelastometry (ROTEM) parameters regarding neonates. This table shows the correlations that were moderate (0.40 ≤ |ρ| ≤ 0.59), strong (0.60 ≤ |ρ| ≤ 0.79) or very strong (0.80 ≤ |ρ|) and with a P-value ≤0.05. 24

Table S16. Regression models for strong and very strong correlations regarding pregnant women with Preeclampsia. The selection of the model was based on the statistical significance of the p-values of the intercept and the coefficients and the adjusted R^2. For each correlation the selected model is highlighted in red. P-values in bold indicate statistical significance at the level α=0.05. 25

Table S17. All logistic regressions with P-value ≤ 0.05 regarding pregnant women with Preeclampsia and neonates born to pregnant women with Preeclampsia.............................27

Table S18. All variables for which a Receiver Operating Characteristic (ROC) curve was created based on the logistic regression's results. P-values in bold indicate statistical significance at the level α=0.05..28

SUPPLEMENTARY TABLES

Table S1. This table shows the Rotational Thromboelastometry (ROTEM) assays, their relevant details (how clot formation is induced in each assay), the ROTEM parameters measured in this study, their respective measurement units, and what each parameter reflects.

ROTEM Assay	Details
**INTEM (Intrinsic ROTEM)**	clot formation is induced by activation of the intrinsic coagulation pathway using 20 µL of 0.2 mol/L calcium chloride solution (star-TEM reagent) and 20 µL of intrinsic activator (in-TEM reagent; partial thromboplastin phospholipid made of rabbit brain and ellagic acid)
**HEPTEM (Heparinase ROTEM)**	a modified INTEM assay, where heparin is degraded by 20 µL of heparin inactivator (hep-TEM reagent; heparinase I from flavobacteria)
**EXTEM (Extrinsic ROTEM)**	clot formation is induced by activation of the extrinsic coagulation pathway using 20 µL of 0.2 mol/L calcium chloride solution (star-TEM reagent) and 20 µL of extrinsic activator (ex-TEM reagent; recombinant tissue factor and phospholipids)
**FIBTEM (Fibrinogen ROTEM)**	a modified EXTEM assay, where platelet contribution to clot formation was inhibited with 20 µL of thrombocyte inhibitor (fib-TEM reagent; Cytochalasin D and 0.2 mol/L of calcium chloride)
**APTEM (Aprotinin ROTEM)**	a modified EXTEM assay, where fibrinolysis was inhibited with 20µL of fibrinolysis inhibitor (ap-TEM reagent; plasmin-antagonist Aprotinin and 0.2 mol/L calcium chloride)

Parameters		Unit	Reflecting
**CT**	Clotting Time	seconds	the time for the formation of a 2mm clot in amplitude
**CFT**	Clot Formation Time	seconds	the time from CT until the achievement of a clot firmness of 20mm
**A-angle**		degrees	the angle between the center line and the tangent to the clotting curve at the 2-mm amplitude point
**CFR**	Clot Formation Rate	degrees	the angle between the baseline and the tangent at the maximum slope
**A10,30**	Amplitude 10,30	millimeters	the amplitude of the clot at 10 and 30 minutes after CT
**MCF**	Maximum Clot Firmness	millimeters	the final amplitude (strength) of the clot
**MCE**	Maximum Clot Elasticity	dynes/cm^2	mechanical properties of the clot
**LI60**	Lysis Index 60	percentage	the percentage of the residual clot firmness compared to the MCF value at 60 minutes after CT
**ML**	Maximum Lysis	percentage	the percentage of the lost clot firmness compared to MCF value
**ACF**	Actual Clot Firmness	millimeters	the remaining firmness of the clot

Table S2. Maternal and neonatal blood count and biochemical tests' results (Preeclampsia Group and Controls). Summary measures are expressed as "Mean ± SD – Standard Deviation" or "Median (IQR – Interquartile Range)" for numerical variables and as Numbers with their respective percentages (%) for categorical variables. P-values in bold indicate statistical significance at the level α=0.05.

	**PE[1] Group (N=31)**	**Pregnant Controls (N=45)**	**P-Value**	**Neonates born to PE mothers (N=34)**	**Neonates born to pregnant controls (N=47)**	**P-Value**
RBC[2] ($x10^6$/μL)	3.9±0.4	3.9±0.4	0.93	4.7±0.7	4.6±0.7	0.75
Hematocrit (%)	35±3.9	34.8±3.1	0.80	52.1±6.5	49.3±7.9	0.13
Hemoglobulin (g/dL)	11.8±1.3	11.7±1	0.66	17.4 (16-18.8)	17.1 (15.1-18)	0.25
WBC[3] ($x10^6$/μL)	11.8 (9.6-13.6)	10.8 (8.7-12.6)	0.32	9.6 (6.5-14.7)	12.8 (10.2-15.8)	**0.02**
Neutrophils (%)	77.6±6.7	74.3±7.3	0.07	39±16.3	47.7±14.3	**0.03**
Lymphocytes (%)	15±5.8	17.1±6.2	0.19	47.8±17.3	36.2±14.8	**0.007**
Creatinine (mg/dL)	0.72±0.12	0.55±0.08	**<0.001**	0.69±0.13	0.6±0.13	**0.04**
Urea (mg/dL)	22 (17-27)	14 (12-16)	**<0.001**	31.4±14.7	19.5±8.3	**0.004**
AST[4] (U/L)	37 (32.5-98.5)	17 (15-21)	**<0.001**	42 (36-57)	45 (38-54)	1.00
ALT[5] (U/L)	56 (46.5-148.5)	12 (8-18)	**<0.001**	7 (5-9.8)	10 (6-12)	0.08
gGT[6] (U/L)	20 (12.5-25)	-	-	111 (60.3-182.5)	113 (85.3-201.3)	0.81
ALP[7] (U/L)	130 (93-152)	123.5 (97.3-156.3)	0.94	189.4±60.7	181.2±51.3	0.67
CRP[8] (mg/dL)	0.8 (0.38-1.21)	0.47 (0.14-1.05)	0.26	0.18 (0.07-0.42)	0.12 (0.04-0.43)	0.43
LDH[9] (U/L)	257 (241-316)	201 (185-213)	**<0.001**	-	-	-
24-Hour Urine Protein (mg/24h)	551.8 (475-2627.1)	-	-	-	-	-

[1]PE: Preeclampsia; [2]RBC: Red Blood Cells; [3]WBC: White Blood Cells; [4]AST: Aspartate Transaminase; [5]ALT: Alanine Transaminase; [6]gGT: gamma-Glutamyltransferase; [7]ALP: Alkaline Phosphatase; [8]CRP: C-Reactive Protein; [9]LDH: Lactate dehydrogenase

Table S3. Baseline characteristics of pregnant women regarding Preeclampsia's severity (Non-Severe Preeclampsia subgroup, Severe Preeclampsia subgroup and Controls). Summary measures are expressed as "Mean ± SD – Standard Deviation" or "Median (IQR – Interquartile Range)" for numerical variables and as Numbers with their respective percentages (%) for categorical variables. P-values in bold indicate statistical significance at the level α=0.05.

PREGNANT WOMEN	Non-Severe PE[1] (N=11)	Severe PE (N=11)	Pregnant Controls (N=19)	P-Value			
				Across Subgroups	Non-Severe PE VS Severe PE	Non-Severe PE VS Controls	Severe PE VS Controls
Age (years)	35.9±5.5	35±5.2	32.2±5.6	0.42	-	-	-
Age above 40	3/11 (27.3%)	1/11 (9.1%)	2/18 (11.1%)	0.53	-	-	-
Race	All Caucasian	All Caucasian	All Caucasian	-	-	-	-
BMI[2] before (kg/m^2)	25.1 (22.3-28.7)	26.4 (24.5-27.6)	22.6 (21.2-25.9)	0.36	-	-	-
BMI at Labor (kg/m^2)	30±2.6	29.3±3	27.8±4.4	0.25	-	-	-
BMI difference (kg/m^2)	4.1 (2.7-5.1)	3.6 (1.7-5.3)	2.8 (1.8-3.8)	0.57	-	-	-
Smoking before pregnancy	2/11 (18.2%)	2/11 (18.2%)	7/19 (36.8%)	0.48	-	-	-
Smoking during pregnancy	1/11 (9.1%)	0/11 (0%)	5/19 (26.3%)	0.14	-	-	-
**Obstetric history**							
Gravidity	2 (1-3)	2 (1-2)	2 (1-3)	0.45	-	-	-
Parity	1 (1-2)	1 (1-2.5)	2 (1-2)	0.36	-	-	-
PE in previous pregnancies	1/11 (9.1%)	1/11 (9.1%)	2/19 (10.5%)	1.00	-	-	-
Pregnancy losses	0 (0-1)	0 (0-0.5)	0 (0-1)	0.43	-	-	-
**Drugs during pregnancy**							
Aspirin	8/11 (72.7%)	6/11 (54.6%)	11/19 (57.9%)	0.72	-	-	-
LMWH[3]	4/11 (36.4%)	3/11 (27.3%)	6/19 (31.6%)	1.00	-	-	-
Progesterone	2/11 (18.2%)	2/11 (18.2%)	3/19 (15.8%)	1.00	-	-	-
Thyroxine	3/11 (27.3%)	3/11 (27.3%)	4/19 (21.1%)	1.00	-	-	-
Steroids	9/11 (81.8%)	8/11 (72.7%)	11/19 (57.9%)	0.40	-	-	-
**Comorbidities and major complications**							
Hypothyroidism	3/11 (27.3%)	3/11 (27.3%)	4/19 (21.1%)	1.00	-	-	-
Hypercholesterolemia	0/11 (0%)	0/11 (0%)	0/19 (0%)	-	-	-	-
Persistent thrombocytopenia after delivery	0/11 (0%)	3/11 (27.3%)	0/19 (0%)	**0.03**	0.64	1.00	0.12
Transfusion	0/11 (0%)	5/11 (45.5%)	0/19 (0%)	**0.001**	0.11	1.00	**0.01**
ICU[4] admission	0/11 (0%)	1/11 (9.1%)	0/19 (0%)	0.54	-	-	-
Death	0/11 (0%)	0/11 (0%)	0/18 (0%)	-	-	-	-
**PE type**							
Early-onset PE	8/11 (72.7%)	9/11 (81.8%)	-	1.00	-	-	-
Late-onset PE	3/11 (27.3%)	2/11 (18.2%)	-	1.00	-	-	-
HELLP[5] syndrome	-	2/11 (18.2%)	-	-	-	-	-

[1]PE: Preeclampsia; [2]BMI: Body Mass Index; [3]LMWH: Low Molecular Weight Heparin; [4]ICU: Intensive Care Unit; [5]HELLP: Hemolysis, Elevated Liver enzymes, Low Platelets syndrome

Table S4. Maternal blood count and maternal biochemical tests' results regarding Preeclampsia's severity (Non-Severe Preeclampsia subgroup, Severe Preeclampsia subgroup and Controls). Summary measures are expressed as "Mean ± SD – Standard Deviation" or "Median (IQR – Interquartile Range)" for numerical variables and as Numbers with their respective percentages (%) for categorical variables. P-values in bold indicate statistical significance at the level α=0.05.

PREGNANT WOMEN	Non-Severe PE[1] (N=11)	Severe-PE (N=11)	Pregnant Controls (N=19)	P-Value			
				Across Subgroups	Non-Severe PE VS Severe PE	Non-Severe PE VS Controls	Severe PE VS Controls
RBC[2] (x10^6/μL)	3.9±0.3	3.8±0.4	3.8±0.3	0.81	-	-	-
Hematocrit (%)	35.7±3	35±3.2	34.2±3.6	0.56	-	-	-
Hemoglobulin (g/dL)	11.9±1	11.9±1	11.5±1.1	0.47	-	-	-
WBC[3] (x10^6/μL)	12.6 (11.6-14.7)	11.4 (10.1-13.9)	11.6 (9.6-13.1)	0.37	-	-	-
Neutrophils (%)	80.4±6.3	78.4±6.1	74.9±7.7	0.15	-	-	-
Lymphocytes (%)	12.3±4.6	14.7±4.9	17.2±7.2	0.14	-	-	-
Creatinine (mg/dL)	0.74±0.11	0.76±0.12	0.57±0.07	**<0.001**	0.85	**<0.001**	**<0.001**
Urea (mg/dL)	28.4±12.6	22.2±9.8	14.9±3.4	**0.005**	0.42	**0.01**	0.09
AST[4] (U/L)	35 (19-40)	94 (68.5-144.5)	18 (14.5-22)	**<0.001**	**0.03**	0.22	**<0.001**
ALT[5] (U/L)	53 (18-58)	123 (100-192.5)	14 (7-22)	**<0.001**	0.12	**0.02**	**<0.001**
gGT[6] (U/L)	29.4±14.9	16±5.9	-	**0.04**	-	-	-
ALP[7] (U/L)	119 (113-140)	129 (81.5-146)	118 (97.5-147.5)	0.92	-	-	-
CRP[8] (mg/dL)	0.72 (0.52-1.13)	0.95 (0.43-1.6)	0.52 (0.14-1.44)	0.73	-	-	-
LDH[9] (U/L)	249.5 (237-295.8)	316 (246.5-445)	202 (180.5-215)	**<0.001**	0.88	**0.02**	**<0.001**
24-Hour Urine Protein (mg/24h)	568 (476-2271.7)	581.6 (451-4312.6)	-	0.86	-	-	-

[1]PE: Preeclampsia; [2]RBC: Red Blood Cells; [3]WBC: White Blood Cells; [4]AST: Aspartate Transaminase; [5]ALT: Alanine Transaminase; [6]gGT: gamma-Glutamyltransferase; [7]ALP: Alkaline Phosphatase; [8]CRP: C-Reactive Protein; [9]LDH: Lactate dehydrogenase

Table S5. Maternal results regarding Preeclampsia's severity (Non-Severe Preeclampsia subgroup, Severe Preeclampsia subgroup and Controls). Summary measures are expressed as "Mean ± SD – Standard Deviation" or "Median (IQR – Interquartile Range)" for numerical variables and as Numbers with their respective percentages (%) for categorical variables. P-values in bold indicate statistical significance at the level α=0.05.

PREGNANT WOMEN		Non-Severe PE[1] (N=11)	Severe-PE (N=11)	Pregnant Controls (N=19)	P-Value			
					Across Subgroups	Non-Severe PE VS Severe PE	Non-Severe PE VS Controls	Severe PE VS Controls
Platelet Count x10^9/L		182.6±40.8	127.2±46.4	225.4±44.3	**<0.001**	**0.02**	**0.04**	**<0.001**
MPV[2] (fL)		9.7 (8.9-10.1)	10.3 (9.5-11.2)	10.2 (9.2-10.6)	0.53	-	-	-
Platelet Count <100x10^9/L		0/11 (0%)	3/11 (27.3%)	0/19 (0%)	**0.03**	0.64	1.00	0.12
Platelet Count <150x10^9/L		2/11 (18.2%)	8/11 (72.7%)	1/19 (5.3%)	**<0.001**	0.09	1.00	**<0.001**
Antithrombin III (%)		89.7±10	94.7±17.4	98.2±11.1	0.30	-	-	-
Protein C activity (%)		93 (83-110)	106 (85.5-107.5)	103 (97-111)	0.57	-	-	-
Free Protein S Ag (%)		32.1±4	23.3±1.7	39.9±5.5	**<0.001**	**<0.001**	**0.002**	**<0.001**
CCTs[3]	PT[8] (seconds)	12.4±0.7	12.6±0.8	13±0.9	0.11	-	-	-
	APTT[9] (seconds)	28.2±3	29±3.4	28.3±1.8	0.73	-	-	-
	INR[10]	0.91 (0.9-0.92)	0.94 (0.9-0.96)	0.97 (0.93-0.99)	**0.03**	1.00	**0.03**	0.41
	Fibrinogen (mg/dL)	465 (431.5-521)	488 (411-658)	455 (448.5-493.5)	0.59	-	-	-
	D-Dimers (ng/mL)	2780 (1785-3405)	2340 (1385-2600)	870 (460-1090)	**0.003**	1.00	**0.01**	**0.02**
INTEM[4]	CT[11] (seconds)	160.8±33.3	167.8±25.6	174.7±26.4	0.43	-	-	-
	CFT[12] (seconds)	61 (52-63.5)	60 (51-104.5)	64 (58.5-67.5)	0.52	-	-	-
	MCF[13] (mm)	71 (69.5-72.5)	73 (68-74.5)	70 (66.5-71.5)	0.22	-	-	-
	A-angle (° degrees)	78 (77-79)	79 (75.5-80)	77 (76-78)	0.28	-	-	-
	A10[14] (mm)	63 (60.5-65.5)	66 (57.5-67.5)	62 (60-62.5)	0.34	-	-	-
	A30[14] (mm)	70 (68.5-71.5)	73 (67.5-74.5)	70 (66-71)	0.29	-	-	-
	MCE[15] (dynes/cm^2)	241 (227-259.5)	265 (217.5-291.5)	231 (198.5-246.5)	0.29	-	-	-
	LI60[16] (%)	97 (96.5-99)	99 (97.5-100)	97 (94.5-97.5)	0.06	-	-	-
	ML[17] (%)	5±3.7	4.3±2.9	6.3±4	0.31	-	-	-
	CFR[18] (° degrees)	79 (78.5-80.5)	81 (80-81.5)	79 (78-79.5)	0.06	-	-	-
	ACF[19] (mm)	67 (65-69.5)	68 (65-71.5)	65 (62-68.5)	0.32	-	-	-
EXTEM[5]	CT (seconds)	55 (52.5-58)	60 (54-63)	61 (52.5-64.5)	0.28	-	-	-
	CFT (seconds)	70 (47.5-75.5)	71 (52.5-124)	74 (59.5-80)	0.39	-	-	-
	MCF (mm)	68 (66.5-71)	69 (65-74)	71 (67-72)	0.81	-	-	-
	A-angle (° degrees)	77 (75-78.8)	79 (67.5-80)	76 (74-78)	0.71	-	-	-
	A10 (mm)	61 (59.5-65.5)	64 (52-67.5)	62 (58.5-63.5)	0.90	-	-	-
	A30 (mm)	68 (66.5-70)	69 (63.5-73.5)	70 (67-72)	0.82	-	-	-
	MCE (dynes/cm^2)	210 (198.5-245)	224 (184-286.5)	240 (204-260.5)	0.81	-	-	-
	LI60 (%)	91 (89-96.5)	96 (89.5-100)	97 (95-97.5)	0.33	-	-	-
	ML (%)	11.5±7.3	9.4±7.9	7.8±4.2	0.33	-	-	-
	CFR (° degrees)	77 (76-79.8)	80 (69-81)	77 (75-79)	0.77	-	-	-
	ACF (mm)	59 (56-64)	62 (57-66)	65 (60.5-67.5)	0.21	-	-	-
FIBTEM[6]	CT (seconds)	51 (48-57.5)	55 (51-57.5)	57 (50.5-61)	0.70	-	-	-
	CFT (seconds)	-	-	-	-	-	-	-
	MCF (mm)	25.7±7.2	27.6±7.1	5.3±24	0.25	-	-	-
	A-angle (° degrees)	75.2±6	75.5±4.1	75.2±3.5	0.98	-	-	-
	A10 (mm)	23.2±7.5	24.9±7.1	21.6±5.3	0.40	-	-	-
	A30 (mm)	25.6±7.5	27.4±7.1	23.5±5.4	0.28	-	-	-
	MCE (dynes/cm^2)	35.9±13.6	39±13.4	31.6±9.3	0.25	-	-	-

	LI60 (%)	99 (98.5-100)	100 (99-100)	100 (100-100)	0.29	-	-	-
	ML (%)	1 (0-2)	1 (0-2.5)	0 (0-1)	0.33	-	-	-
	CFR (° degrees)	77 (75-79)	79 (75-80)	77 (75-79)	0.50	-	-	-
	ACF (mm)	25.5±6.9	27.5±6.9	24±4.7	0.31	-	-	-
**APTEM[7]**	CT (seconds)	51 (48-55)	56 (54-60.5)	60 (53.5-71.5)	**0.02**	0.12	**0.02**	1.00
	CFT (seconds)	65 (54.5-75.5)	70 (52.5-135.5)	81 (66-86.5)	0.32	-	-	-
	MCF (mm)	70 (67-73)	72 (59.5-74.5)	68 (65-70)	0.29	-	-	-
	A-angle (° degrees)	78 (75.5-79.5)	79 (72.5-80)	74 (72.5-76)	0.07	-	-	-
	A10 (mm)	62 (56.5-65.5)	63 (47-67)	59 (55.5-60)	0.32	-	-	-
	A30 (mm)	70 (65.5-73)	72 (58-74.5)	68 (64.5-69)	0.32	-	-	-
	MCE (dynes/cm^2)	242.6±48.3	222±83.9	206.8±53.5	0.21	-	-	-
	LI60 (%)	97 (94-99)	98 (98-99)	97 (97-98)	0.23	-	-	-
	ML (%)	5 (2.5-11)	4 (3-4.5)	6 (3-9.5)	0.21	-	-	-
	CFR (° degrees)	78 (76.5-80)	80 (74.5-81.5)	75 (74-78)	0.07	-	-	-
	ACF (mm)	64 (62.5-68.5)	67 (58-72)	64 (57-65.5)	0.36	-	-	-

[1]PE: Preeclampsia; [2]MPV: Mean Platelet Volume; [3]CCTs: Conventional Coagulation Tests; [4]INTEM: Intrinsic Thromboelastometry; [5]EXTEM: Extrinsic Thromboelastometry; [6]FIBTEM: Fibrinogen Thromboelastometry; [7]APTEM: Aprotinin Thromboelastometry; [8]PT: Prothrombin Time; [9]APTT: Activated Partial Thromboplastin Time; [10]INR: International Normalized Ratio; [11]CT: Clotting Time; [12]CFT: Clot Formation Time; [13]MCF: Maximum Clot Firmness; [14]A10,30: Amplitude at 10, 20, 30 minutes after Clotting Time (post CT); [15]MCE: Maximum Clot Elasticity; [16]LI60: Lysis Index at 60 minutes after Clotting Time (post CT); [17]ML: Maximum Lysis; [18]CFR: Clot Formation Rate; [19]ACF: Actual Clot Firmness

Table S6. Baseline characteristics of neonates regarding Preeclampsia's severity (Non-Severe Preeclampsia subgroup, Severe Preeclampsia subgroup and Controls). Summary measures are expressed as "Mean ± SD – Standard Deviation" or "Median (IQR – Interquartile Range)" for numerical variables and as Numbers with their respective percentages (%) for categorical variables. P-values in bold indicate statistical significance at the level α=0.05.

**NEONATES**	**Neonates born to Non-Severe PE[1] mothers (N=13)**	**Neonates born to Severe-PE mothers (N=13)**	**Neonates born to pregnant controls (N=21)**	**P-Value**			
				**Across Subgroups**	**Non-Severe PE VS Severe PE**	**Non-Severe PE VS Controls**	**Severe PE VS Controls**
GA[2] (weeks)	32.3±3.1	31.3±3.6	31.5±3.6	0.74	-	-	-
Gender (male)	6/13 (46.2%)	7/13 (53.9%)	12/21 (57.1%)	0.82			
Term	0/13 (0%)	0/13 (0%)	0/21 (0%)	-	-	-	-
Preterm	13/13 (100%)	13/13 (100%)	21/21 (100%)	-	-	-	-
Extremely preterm	1/13 (7.7%)	2/13 (15.4%)	3/21 (14.3%)	1.00	-	-	-
Very preterm	3/13 (23.1%)	4/13 (30.8%)	6/21 (28.6%)	1.00	-	-	-
Moderate preterm	5/13 (38.5%)	3/13 (23.1%)	5/21 (23.8%)	0.70	-	-	-
Late preterm	4/13 (30.8%)	4/13 (30.8%)	7/21 (33.3%)	1.00	-	-	-
Birthweight (grams)	1968.1±826	1754.6±818.4	1733.3±839	0.70	-	-	-
Below 1000 grams	2/13 (15.4%)	4/13 (30.8%)	5/21 (23.8%)	0.63	-	-	-
Below 2500 grams	8/13 (61.5%)	11/13 (84.6%)	16/21 (76.2%)	0.40	-	-	-
Birthweight percentile	48.5±29.5	41.7±33.2	42±26	0.79	-	-	-
Length (cm)	42.4±7.1	41.9±6.4	41.7±5.7	0.95	-	-	-
Length percentile	57.7±36.3	51.7±34.2	49.3±30.5	0.77	-	-	-
Head circumference (cm)	31.2±5.5	29.3±3.1	29.5±4.4	0.23	-	-	-
Circumference percentile	71.3±25.4	54.2±24.5	59.9±30.2	0.29	-	-	-
SGA[3]	1/13 (7.7%)	2/13 (15.4%)	2/21 (9.5%)	0.85	-	-	-
IUGR[4]	2/13 (15.4%)	4/13 (30.8%)	5/21 (23.8%)	0.63	-	-	-
**Pregnancy characteristics**							
Twins	1/13 (7.7%)	4/13 (30.8%)	4/21 (19.1%)	0.41	-	-	-
ART[5]	5/13 (38.5%)	5/13 (38.5%)	9/21 (42.9%)	0.96	-	-	-
Mean uterine artery's PI [6]	1 (0.7-1.4)	0.9 (0.9-2.2)	0.7 (0.5-0.7)	**0.04**	1	0.08	0.07
Umbilical artery's PI	1.1±0.2	1±0.1	1±0.1	0.33	-	-	-
**Delivery characteristics**							
Delivery Type (CS[7])	13/13 (100%)	13/13 (100%)	21/21 (100%)	-	-	-	-
Placenta's weight (grams)	420 (320-480)	310 (260-450)	400 (345-440)	0.22	-	-	-
Umbilical cord's clamp (seconds)	60 (40-60)	60 (50-60)	60 (30-60)	0.85	-	-	-
Apgar score at 1 minute	8 (7-8)	7 (7-8)	7 (6-8)	0.44	-	-	-
Apgar score at 5 minutes	8 (8-8)	8 (8-8)	8 (8-9)	0.70	-	-	-
**Neonatal major complications**							
NICU[8] admission	11/13 (84.6%)	11/13 (84.6%)	18/21 (85.7%)	1.00	-	-	-
Respiratory assistance	10/13 (76.9%)	9/13 (69.2%)	18/21 (85.7%)	0.54	-	-	-
Infection	4/13 (30.8%)	5/13 (38.5%)	8/21 (38.1%)	0.93	-	-	-
Sepsis	1/13 (7.7%)	3/13 (23.1%)	2/21 (9.5%)	0.54	-	-	-
RDS[9]	5/13 (38.5%)	7/13 (53.9%)	9/21 (42.9%)	0.71	-	-	-
BPD[10]	2/13 (15.4%)	3/13 (23.1%)	1/21 (4.8%)	0.39	-	-	-
IVH[11]	1/13 (7.7%)	1/13 (7.7%)	2/21 (9.5%)	1.00	-	-	-
NEC[12]	0/13 (0%)	2/13 (15.4%)	1/21 (4.8%)	0.44	-	-	-
Thrombocytopenia	0/13 (0%)	3/13 (23.1%)	1/21 (4.8%)	0.15	-	-	-
Transfusion	2/13 (15.4%)	4/13 (30.8%)	9/21 (42.9%)	0.30	-	-	-
Death	0/13 (0%)	1/13 (7.7%)	0/21 (0%)	0.55	-	-	-

[1]PE: Preeclampsia; [2]GA: Gestational Age at birth; [3]SGA: Small for Gestational Age; [4]IUGR: Intrauterine Growth Restriction; [5]ART: Assisted Reproductive Technology; [6]PI: Pulsatility Index; [7]CS: Caesarian Section; [8]NICU: Neonatal Intensive Care Unit; [9]RDS: Respiratory Distress Syndrome; [10]BPD: Bronchopulmonary Dysplasia; [11]IVH: Intraventricular Hemorrhage; [12]NEC: Necrotizing Enterocolitis

Table S7. Neonatal blood count and neonatal biochemical tests' results regarding Preeclampsia's severity (Non-Severe Preeclampsia subgroup, Severe Preeclampsia subgroup and Controls). Summary measures are expressed as "Mean ± SD – Standard Deviation" or "Median (IQR – Interquartile Range)" for numerical variables and as Numbers with their respective percentages (%) for categorical variables. P-values in bold indicate statistical significance at the level α=0.05.

**NEONATES**	**Neonates born to Non-Severe -PE[1] mothers (N=13)**	**Neonates born to Severe-PE mothers (N=13)**	**Neonates born to pregnant controls (N=21)**	**P-Value**			
				**Across Subgroups**	**Non-Severe PE VS Severe PE**	**Non-Severe PE VS Controls**	**Severe PE VS Controls**
RBC[2] (x10^6/μL)	4.5±0.6	4.7±0.8	4.2±0.8	0.20	-	-	-
Hematocrit (%)	50.4±4.8	53.7±7.5	46.4±10.7	0.18	-	-	-
Hemoglobulin (g/dL)	16.8 (15.5-17.6)	18.7 (17.7-19.3)	14.1 (13.5-17.2)	**0.03**	0.35	0.68	**0.02**
WBC[3] (x10^6/μL)	10.3±5.3	7.9±3.7	11.5±5.4	0.19	-	-	-
Neutrophils (%)	33.8±14.3	36.4±17.8	45.5±17.5	0.21	-	-	-
Lymphocytes (%)	54.3±15.8	49.9±17.9	39.7±19	0.13	-	-	-
Creatinine (mg/dL)	0.72±0.13	0.63±0.13	0.61±0.13	0.14	-	-	-
Urea (mg/dL)	34.3±16.1	27.2±12.3	23.8±7.7	0.18	-	-	-
AST[4] (U/L)	42 (40-50)	47 (33-92.3)	51.5 (37.5-54.3)	0.98	-	-	-
ALT[5] (U/L)	8 (5-11)	6.5 (5-8.3)	5.5 (5-8.5)	0.64	-	-	-
gGT[6] (U/L)	119 (104-214)	86.5 (52-125.3)	97.5 (71.5-132.5)	0.21	-	-	-
ALP[7] (U/L)	161.9±54.7	208.5±56.2	184.8±49.9	0.13	-	-	-
CRP[8] (mg/dL)	0.13 (0.08-0.42)	0.21 (0.04-0.48)	0.07 (0.03-0.21)	0.31	-	-	-

[1]PE: Preeclampsia; [2]RBC: Red Blood Cells; [3]WBC: White Blood Cells; [4]AST: Aspartate Transaminase; [5]ALT: Alanine Transaminase; [6]gGT: gamma-Glutamyltransferase; [7]ALP: Alkaline Phosphatase; [8]CRP: C-Reactive Protein

Table S8. Baseline characteristics of pregnant women when divided into Early- and Late-Onset Preeclampsia subgroups (Early-Onset Preeclampsia subgroup, Late-Onset Preeclampsia subgroup and Controls). Summary measures are expressed as "Mean ± SD – Standard Deviation" or "Median (IQR – Interquartile Range)" for numerical variables and as Numbers with their respective percentages (%) for categorical variables. P-values in bold indicate statistical significance at the level α=0.05.

PREGNANT WOMEN	Early-Onset PE[1] (N=19)	Late-Onset PE (N=12)	Pregnant Controls (N=32)	P-Value			
				Across Subgroups	Early-Onset PE VS Late-Onset PE	Early-Onset PE VS Controls	Late-Onset PE VS Controls
Age (years)	35 (31-39)	33 (31.8-34.3)	35 (28.8-37)	0.28	-	-	-
Age above 40	4/19 (21.1%)	0/12 (0%)	4/32 (12.5%)	0.24	-	-	-
Race	All Caucasian	All Caucasian	All Caucasian	-	-	-	-
BMI[2] before (kg/m^2)	26.4 (23.7-28.2)	26.3 (21.6-28.6)	23.9 (21.4-26.4)	0.18	-	-	-
BMI at Labor (kg/m^2)	29.8 (28.8-32)	29.6 (28.9-31.1)	28.7 (25.8-31.6)	0.35	-	-	-
BMI difference (kg/m^2)	3.6±2.1	3.4±2.7	4±2.4	0.74	-	-	-
Smoking before pregnancy	4/19 (21.1%)	3/12 (25%)	10/32 (31.3%)	0.81	-	-	-
Smoking during pregnancy	1/19 (5.3%)	1/12 (8.3%)	6/32 (18.8%)	0.47	-	-	-
**Obstetric history**							
Gravidity	2 (1-2.5)	2 (1-3)	2 (1-3)	0.62	-	-	-
Parity	1 (1-2)	1.5 (1-2.3)	2 (1-2)	0.30	-	-	-
PE in previous pregnancies	2/19 (10.5%)	1/12 (8.3%)	2/32 (6.3%)	0.84	-	-	-
Pregnancy losses	0 (0-0.5)	0 (0-0.3)	0 (0-1)	0.36	-	-	-
**Drugs during pregnancy**							
Aspirin	11/19 (57.9%)	10/12 (83.3%)	19/32 (59.4%)	0.32	-	-	-
LMWH[3]	8/19 (42.1%)	4/12 (33.3%)	11/32 (34.4%)	0.83	-	-	-
Progesterone	4/19 (21.1%)	2/12 (16.7%)	8/32 (25%)	0.92	-	-	-
Thyroxine	8/19 (42.1%)	6/12 (50%)	13/32 (40.6%)	0.85	-	-	-
Steroids	14/19 (73.7%)	7/12 (58.3%)	19/32 (59.4%)	0.58	-	-	-
**Comorbidities and major complications**							
Hypothyroidism	8/19 (42.1%)	6/12 (50%)	13/32 (40.6%)	0.85	-	-	-
Hypercholesterolemia	0/19 (0%)	1/12 (8.3%)	2/32 (6.3%)	0.58	-	-	-
Persistent thrombocytopenia after delivery	2/19 (10.5%)	1/12 (8.3%)	0/32 (0%)	0.11	-	-	-
Transfusion	7/19 (36.8%)	0/12 (0%)	8/32 (25%)	**0.045**	0.08	1.00	0.25
ICU[4] admission	1/19 (5.3%)	0/12 (0%)	0/32 (0%)	0.49	-	-	-
Death	0/19 (0%)	0/12 (0%)	0/32 (0%)	-	-	-	-
**PE type**							
Severe PE	9/19 (47.4%)	2/12 (16.7%)	-	0.23	-	-	-
HELLP[5] syndrome	2/19 (10.5%)	0/12 (0%)	-	0.51	-	-	-

[1]PE: Preeclampsia; [2]BMI: Body Mass Index; [3]LMWH: Low Molecular Weight Heparin; [4]ICU: Intensive Care Unit; [5]HELLP: Hemolysis, Elevated Liver enzymes, Low Platelets syndrome

Table S9. Maternal blood count and maternal biochemical tests' results when pregnant women are divided into Early- and Late-Onset Preeclampsia subgroups (Early-Onset Preeclampsia subgroup, Late-Onset Preeclampsia subgroup and Controls). Summary measures are expressed as "Mean ± SD – Standard Deviation" or "Median (IQR – Interquartile Range)" for numerical variables and as Numbers with their respective percentages (%) for categorical variables. P-values in bold indicate statistical significance at the level α=0.05.

**PREGNANT WOMEN**	**Early-Onset PE[1] (N=19)**	**Late-Onset PE (N=12)**	**Pregnant Controls (N=32)**	**P-Value**			
				**Across Subgroups**	**Early-Onset PE VS Late-Onset PE**	**Early-Onset PE VS Controls**	**Late-Onset PE VS Controls**
RBC[2] (x10^6/μL)	3.8±0.4	4.1±0.5	3.8±0.4	**0.043**	**0.04**	0.77	0.12
Hematocrit (%)	34.5±3.3	35.8±4.7	34.8±3.3	0.65	-	-	-
Hemoglobulin (g/dL)	11.7±1.1	12±1.6	11.7±1	0.75	-	-	-
WBC[3] (x10^6/μL)	12.6 (10.5-14.3)	10.3 (9.1-11.6)	10.8 (8.6-12.5)	0.14	-	-	-
Neutrophils (%)	79.7±6.5	74.4±5.8	74.5±7	**0.03**	0.09	**0.04**	0.99
Lymphocytes (%)	10.5 (9.8-15.5)	15.9 (14-19.7)	16 (12.5-21.2)	**0.03**	0.06	0.12	1.00
Creatinine (mg/dL)	0.75±0.12	0.68±0.12	0.55±0.08	**<0.001**	0.11	**<0.001**	**0.002**
Urea (mg/dL)	24 (20.5-31.5)	18.5 (14.8-25.5)	14 (12-16)	**<0.001**	0.43	**<0.001**	**0.04**
AST[4] (U/L)	43 (35-98.5)	34.5 (18.3-100.3)	17 (14.8-20)	**<0.001**	0.55	**<0.001**	**0.03**
ALT[5] (U/L)	60 (52.5-148.5)	53 (22.8-88.5)	11.5 (7.8-15.5)	**<0.001**	0.77	**<0.001**	**0.001**
gGT[6] (U/L)	20 (11-26)	20 (18-22)	-	0.92	-	-	-
ALP[7] (U/L)	112.4±38.2	166.1±77.2	125.2±49.6	**0.04**	**0.03**	0.75	0.12
CRP[8] (mg/dL)	0.84 (0.5-1.2)	0.6 (0.28-1.3)	0.46 (0.14-0.95)	0.42	-	-	-
LDH[9] (U/L)	294 (246-411)	246.5 (211-316)	201 (182.5-213)	**<0.001**	0.70	**<0.001**	**0.045**
24-Hour Urine Protein (mg/24h)	581.6 (449.5-3705.1)	543 (484.7-598.6)	-	0.61	-	-	-

[1]PE: Preeclampsia; [2]RBC: Red Blood Cells; [3]WBC: White Blood Cells; [4]AST: Aspartate Transaminase; [5]ALT: Alanine Transaminase; [6]gGT: gamma-Glutamyltransferase; [7]ALP: Alkaline Phosphatase; [8]CRP: C-Reactive Protein; [9]LDH: Lactate dehydrogenase

Table S10. Maternal results when pregnant women are divided into Early- and Late-Onset Preeclampsia subgroups (Early-Onset Preeclampsia subgroup, Late-Onset Preeclampsia subgroup and Controls). Summary measures are expressed as "Mean ± SD – Standard Deviation" or "Median (IQR – Interquartile Range)" for numerical variables and as Numbers with their respective percentages (%) for categorical variables. P-values in bold indicate statistical significance at the level α=0.05.

**PREGNANT WOMEN**		**Early-Onset PE[1] (N=19)**	**Late-Onset PE (N=12)**	**Pregnant Controls (N=32)**	**P-Value**			
					**Across Subgroups**	**Early-Onset PE VS Late-Onset PE**	**Early-Onset PE VS Controls**	**Late-Onset PE VS Controls**
	Platelet Count $x10^9$/L	156.7±51.3	196.3±84.6	231.8±48.5	**<0.001**	0.16	**<0.001**	0.17
	MPV[2] (fL)	10.1 (9.5-11.2)	9.6 (7.9-10.2)	10.1 (9.3-10.6)	0.36	-	-	-
	Platelet Count <100$x10^9$/L	2/19 (10.5%)	1/12 (8.3%)	0/32 (0%)	0.11	-	-	-
	Platelet Count <150$x10^9$/L	9/19 (47.4%)	3/12 (25%)	1/32 (3.1%)	**<0.001**	0.82	**<0.001**	0.17
	Antithrombin III (%)	88±12.2	100.3±15.9	97.4±11.2	**0.02**	**0.03**	0.06	0.81
	Protein C activity (%)	95 (82-106)	112 (88.5-121)	102.5 (97.3-112.5)	0.16	-	-	-
	Free Protein S Ag (%)	27.3±4.8	30.9±4.6	39.6±6.9	**<0.001**	0.21	**<0.001**	**<0.001**
**CCTs[3]**	PT[8] (seconds)	12.6 (12.1-12.9)	12.4 (12.2-13.5)	12.9 (12.3-13.5)	0.42	-	-	-
	APTT[9] (seconds)	28.8 (26.7-30)	29.8 (27.8-30.3)	28.7 (27.8-29.5)	0.63	-	-	-
	INR[10]	0.92 (0.9-0.94)	0.92 (0.91-0.99)	0.95 (0.93-0.99)	0.18	-	-	-
	Fibrinogen (mg/dL)	497.2±121.1	542±90.1	446.2±115	**0.04**	0.53	0.27	**0.04**
	D-Dimers (ng/mL)	2170 (1865-2600)	1940 (1287.5-2700)	1090 (785-1585)	**<0.001**	1.00	**0.002**	**0.04**
**INTEM[4]**	CT[11] (seconds)	160.9±26.3	167.4±26.9	166±29.5	0.77	-	-	-
	CFT[12] (seconds)	60 (48.5-63.5)	59 (50-68.8)	63 (54-73)	0.58	-	-	-
	MCF[13] (mm)	71 (69-74)	73 (69.8-75)	70 (65-71.3)	0.07	-	-	-
	A-angle (° degrees)	78 (77-80)	79 (75.8-79.5)	77.5 (76-79)	0.29	-	-	-
	A10[14] (mm)	65 (60.5-67)	62 (60-68.3)	61.5 (57-64.3)	0.24	-	-	-
	A30[14] (mm)	71 (68.5-73.5)	71.5 (68.8-75)	69 (65-71.3)	0.14	-	-	-
	MCE[15] (dynes/cm^2)	245.8±70.6	265.8±55.3	229.7±57.8	0.22	-	-	-
	LI60[16] (%)	97 (97-99.8)	99 (97-99.5)	97 (95-98)	0.10	-	-	-
	ML[17] (%)	4.5±3.4	4.1±2.8	6.5±3.7	0.06	-	-	-
	CFR[18] (° degrees)	80 (79-81.5)	80 (77.8-81.3)	79 (77-80)	0.15	-	-	-
	ACF[19] (mm)	67 (63.5-70.5)	70 (67.8-71.3)	64.5 (61-68.3)	**0.02**	0.76	0.30	**0.02**
**EXTEM[5]**	CT (seconds)	55 (52-59.5)	61 (57.5-75.3)	58 (52.8-61)	**0.04**	**0.04**	1.00	0.19
	CFT (seconds)	71 (51.5-84)	57.5 (46.5-64.8)	68 (56.8-80)	0.29	-	-	-
	MCF (mm)	69 (66.5-74)	70 (67.5-75.3)	70.5 (66-73)	0.87	-	-	-
	A-angle (° degrees)	78 (74.5-80)	79 (77-80)	77 (75-79)	0.36	-	-	-
	A10 (mm)	64 (59.5-67.5)	62.5 (57.8-68.5)	62 (57-66)	0.61	-	-	-
	A30 (mm)	68 (66.5-73.5)	69.5 (67-74.3)	70 (66-72.3)	0.95	-	-	-
	MCE (dynes/cm^2)	227±66.6	235.1±92	229.7±54.5	0.95	-	-	-
	LI60 (%)	92 (89-95.8)	96 (94.5-100)	97 (93-98)	0.07	-	-	-
	ML (%)	12 (7-15.5)	5 (2.5-10.5)	7 (5-13)	0.11	-	-	-
	CFR (° degrees)	78 (76-81)	80 (78.5-81)	78 (76-80)	0.41	-	-	-
	ACF (mm)	61 (57-65)	63 (57.8-69)	64 (59.3-67)	0.41	-	-	-
**FIBTEM[6]**	CT (seconds)	51 (48.5-57.5)	54 (51.3-59)	57.5 (53.5-70)	0.07	-	-	-
	CFT (seconds)	-	-	-	-	-	-	-
	MCF (mm)	27.5±7.3	27.8±8.2	23.4±5.7	**0.04**	0.99	0.09	0.13
	A-angle (° degrees)	76 (73-79)	78 (75.5-79.5)	74 (71-77)	**0.04**	0.46	0.08	**0.03**
	A10 (mm)	25.2±7.2	25.4±8.6	21.1±5.5	0.06	-	-	-
	A30 (mm)	27.4±7.3	27.8±8.4	23.3±5.8	0.05	-	-	-
	MCE (dynes/cm^2)	39.2±14.3	40.8±18.1	31.1±10	0.06	-	-	-

	LI60 (%)	99.5 (99-100)	100 (99.5-100)	100 (99-100)	0.53	-	-	-
	ML (%)	1 (0-2.5)	0.5 (0-2)	0 (0-2)	0.30	-	-	-
	CFR (° degrees)	78 (75-80.5)	79 (76-80.5)	75 (72-78)	**0.03**	0.68	**0.03**	**0.03**
	ACF (mm)	27.2±6.9	28.1±7.6	23.6±5.3	0.05	-	-	-
**APTEM[7]**	CT (seconds)	54 (50.5-55.5)	57.5 (51-62)	58.5 (53-67)	0.09	-	-	-
	CFT (seconds)	69 (52.5-96.5)	69 (57.5-125)	79 (66-89.8)	0.30	-	-	-
	MCF (mm)	72 (66.5-73.5)	71 (66.8-74.5)	68 (64-70.3)	0.07	-	-	-
	A-angle (° degrees)	78 (74.5-80)	79 (76-80)	74 (72-77)	**0.008**	1.00	0.05	**0.03**
	A10 (mm)	63 (56.5-67)	60.5 (53.8-65.5)	57 (53.8-61)	0.08	-	-	-
	A30 (mm)	71 (65.5-73.5)	70 (64.8-74.3)	67 (62.8-69.3)	0.08	-	-	-
	MCE (dynes/cm^2)	237.8±72.4	234.6±80.8	204.3±54.8	0.16	-	-	-
	LI60 (%)	98 (97-98.8)	99 (98-100)	98 (97-99)	0.24	-	-	-
	ML (%)	4 (3-6.5)	4.5 (3.5-9.3)	6 (3-9)	0.43	-	-	-
	CFR (° degrees)	78 (76-81)	80 (76.8-81.3)	75.5 (73.8-78)	**0.002**	0.86	0.05	**0.004**
	ACF (mm)	66 (61.5-71.5)	67.5 (60.3-70.3)	62 (57.8-67)	0.13	-	-	-

[1]PE: Preeclampsia; [2]MPV: Mean Platelet Volume; [3]CCTs: Conventional Coagulation Tests; [4]INTEM: Intrinsic Thromboelastometry; [5]EXTEM: Extrinsic Thromboelastometry; [6]FIBTEM: Fibrinogen Thromboelastometry; [7]APTEM: Aprotinin Thromboelastometry; [8]PT: Prothrombin Time; [9]APTT: Activated Partial Thromboplastin Time; [10]INR: International Normalized Ratio; [11]CT: Clotting Time; [12]CFT: Clot Formation Time; [13]MCF: Maximum Clot Firmness; [14]A10,30: Amplitude at 10, 20, 30 minutes after Clotting Time (post CT); [15]MCE: Maximum Clot Elasticity; [16]LI60: Lysis Index at 60 minutes after Clotting Time (post CT); [17]ML: Maximum Lysis; [18]CFR: Clot Formation Rate; [19]ACF: Actual Clot Firmness

Table S11. Baseline characteristics of neonates regarding Preeclampsia's onset (Early-Onset Preeclampsia subgroup, Late-Onset Preeclampsia subgroup and Controls). Summary measures are expressed as "Mean ± SD – Standard Deviation" or "Median (IQR – Interquartile Range)" for numerical variables and as Numbers with their respective percentages (%) for categorical variables. P-values in bold indicate statistical significance at the level α=0.05.

**NEONATES**	**Neonates born to Early-Onset PE[1] mothers (N=21)**	**Neonates born to Late-Onset PE mothers (N=13)**	**Neonates born to pregnant controls (N=34)**	**P-Value**			
				**Across Subgroups**	**Early-Onset PE VS Late-Onset PE**	**Early-Onset PE VS Controls**	**Late-Onset PE VS Controls**
GA[2] (weeks)	31 (28-33)	36 (36-37)	32 (30.3-36)	**<0.001**	**<0.001**	0.06	**0.02**
Gender (male)	8/21 (38.1%)	9/13 (69.2%)	19/34 (55.9%)	0.19	-	-	-
Term	0/21 (0%)	6/13 (46.2%)	8/34 (23.5%)	**0.002**	**0.004**	0.06	0.49
Preterm	21/21 (100%)	7/13 (53.8%)	26/34 (76.5%)	**0.002**	**0.004**	0.06	0.49
Extremely preterm	4/21 (19%)	0/7 (0%)	4/26 (15.3%)	0.67	-	-	-
Very preterm	8/21 (38.1%)	0/7 (0%)	8/26 (30.8%)	0.16	-	-	-
Moderate preterm	8/21 (38.1%)	0/7 (0%)	8/26 (30.8%)	0.16	-	-	-
Late preterm	1/21 (4.8%)	7/7 (100%)	6/26 (23.1%)	**<0.001**	**<0.001**	0.35	**0.001**
Birthweight (grams)	1600 (830-1960)	2940 (2690-3150)	1615 (1252.5-3027.5)	**<0.001**	**<0.001**	0.34	**0.02**
Below 1000 grams	7/21 (33.3%)	0/13 (0%)	5/34 (14.7%)	**0.04**	0.09	0.53	0.91
Below 2500 grams	19/21 (90.5%)	3/13 (23.1%)	23/34 (67.7%)	**<0.001**	**<0.001**	0.30	**0.03**
Birthweight percentile	39 (22-64)	53 (33-73)	34.5 (24.2-68.5)	0.49	-	-	-
Length (cm)	39±6.4	48.9±2	43.2±6.3	**<0.001**	**<0.001**	0.054	**<0.001**
Length percentile	34 (14-76)	80 (62.5-94.9)	45.4 (23.9-80.9)	0.06	-	-	-
Head circumference (cm)	30 (26-31)	33.5 (33-34)	30.3 (28.1-33)	**0.002**	**0.002**	0.98	**0.01**
Circumference percentile	58.6±26.5	62.5±29.5	53.6±27.8	0.59	-	-	-
SGA[3]	3/21 (14.3%)	1/13 (7.7%)	4/34 (11.8%)	1.00	-	-	-
IUGR[4]	7/21 (33.3%)	1/13 (7.7%)	8/34 (23.5%)	0.24	-	-	-
Pregnancy characteristics							
Twins	4/21 (19.1%)	2/13 (15.4%)	4/34 (11.8%)	0.75	-	-	-
ART[5]	9/21 (42.9%)	3/13 (23.1%)	11/34 (32.4%)	0.49	-	-	-
Mean uterine artery's PI[6]	1.2 (0.8-2.1)	0.8 (0.7-1)	0.7 (0.5-0.8)	**0.007**	0.49	**0.006**	1.00
Umbilical artery's PI	1±0.2	1.1±0.2	1.1±0.1	0.78	-	-	-
Delivery characteristics							
Delivery Type (CS[7])	21/21 (100%)	12/13 (92.3%)	31/34 (91.2%)	0.40	-	-	-
Placenta's weight (grams)	320 (300-380)	500 (450-520)	400 (372.5-490)	**<0.001**	**<0.001**	**0.03**	**0.03**
Umbilical cord's clamp (seconds)	60 (40-60)	60 (60-60)	60 (40-60)	0.60	-	-	-
Apgar score at 1 minute	7 (7-8)	8 (8-8)	7.5 (7-8)	**0.02**	**0.02**	1.00	0.06
Apgar score at 5 minutes	8 (8-8)	9 (8-9)	9 (8-9)	**0.006**	**0.007**	0.053	0.60
Neonatal major complications							
NICU[8] admission	21/21 (100%)	3/13 (23.1%)	23/34 (67.7%)	**<0.001**	**<0.001**	**0.01**	**0.03**
Respiratory assistance	18/21 (85.7%)	3/13 (23.1%)	25/34 (73.5%)	**<0.001**	**0.002**	1.00	**0.008**
Infection	8/21 (38.1%)	3/13 (23.1%)	9/34 (26.5%)	0.60	-	-	-
Sepsis	3/21 (14.3%)	1/13 (7.7%)	2/34 (5.9%)	0.56	-	-	-
RDS[9]	13/21 (61.9%)	0/13 (0%)	14/34 (41.2%)	**0.002**	**0.004**	0.67	**0.049**
BPD[10]	5/21 (23.8%)	0/13 (0%)	2/34 (5.9%)	0.054	-	-	-
IVH[11]	2/21 (9.5%)	0/13 (0%)	2/34 (5.9%)	0.66	-	-	-
NEC[12]	2/21 (9.5%)	0/13 (0%)	1/34 (2.9%)	0.43	-	-	-
Thrombocytopenia	3/21(14.3%)	0/13 (0%)	1/34 (2.9%)	0.18	-	-	-
Transfusion	7/21 (33.3%)	0/13 (0%)	10/34 (29.4%)	**0.049**	0.09	1.00	0.13
Death	1/21 (4.8%)	0/13 (0%)	0/34 (0%)	0.50	-	-	-

[1]PE: Preeclampsia; [2]GA: Gestational Age at birth; [3]SGA: Small for Gestational Age; [4]IUGR: Intrauterine Growth Restriction; [5]ART: Assisted Reproductive Technology; [6]PI: Pulsatility Index; [7]CS: Caesarian Section; [8]NICU: Neonatal Intensive Care Unit; [9]RDS: Respiratory Distress Syndrome; [10]BPD: Bronchopulmonary Dysplasia; [11]IVH: Intraventricular Hemorrhage; [12]NEC: Necrotizing Enterocolitis

Table S12. Neonatal blood count and neonatal biochemical tests' results regarding Preeclampsia's onset (Early-Onset Preeclampsia subgroup, Late-Onset Preeclampsia subgroup and Controls). Summary measures are expressed as "Mean ± SD – Standard Deviation" or "Median (IQR – Interquartile Range)" for numerical variables and as Numbers with their respective percentages (%) for categorical variables. P-values in bold indicate statistical significance at the level α=0.05.

NEONATES	Neonates born to Early-Onset PE[1] mothers (N=21)	Neonates born to Late-Onset PE mothers (N=13)	Neonates born to pregnant controls (N=34)	P-Value			
				Across Subgroups	Early-Onset PE VS Late-Onset PE	Early-Onset PE VS Controls	Late-Onset PE VS Controls
RBC[2] (x10^6/μL)	4.4±0.7	5.1±0.5	4.6±0.7	**0.041**	**0.03**	0.67	0.21
Hematocrit (%)	51.2±7	53.8±5.3	50.8±7.8	0.47	-	-	-
Hemoglobulin (g/dL)	16.9 (15.2-18.8)	18.1 (16.9-20.2)	17.3 (15.9-18.8)	0.38	-	-	-
WBC[3] (x10^6/μL)	6.7 (5.1-9.6)	14.4 (12.3-15.6)	12.8 (8.6-17.1)	**<0.001**	**0.003**	**0.01**	1.00
Neutrophils (%)	34.8±16.7	46.3±13.2	46.8±16.5	**0.04**	0.12	0.07	1.00
Lymphocytes (%)	52.3±17.8	39.9±13.6	38.1±16.8	**0.03**	0.11	**0.04**	0.96
Creatinine (mg/dL)	0.73 (0.62-0.8)	0.72 (0.62-0.77)	0.58 (0.52-0.64)	0.24	-	-	-
Urea (mg/dL)	35 (26-40)	20 (12.8-30.5)	23 (16.3-27)	**0.007**	0.07	**0.02**	1.00
AST[4] (U/L)	42 (36-77)	39 (36.3-49.3)	51.5 (38.8-56.5)	0.50	-	-	-
ALT[5] (U/L)	5 (5-7)	9 (9-11)	9 (5.8-15)	**0.01**	**0.02**	0.10	1.00
gGT[6] (U/L)	108 (58-170.5)	119 (104-336)	127 (96-179)	0.62	-	-	-
ALP[7] (U/L)	192.6±60.4	175.8±67.2	192.1±51.3	0.84	-	-	-
CRP[8] (mg/dL)	0.2 (0.05-0.42)	0.14 (0.11-0.36)	0.11 (0.03-0.21)	0.45	-	-	-

[1]PE: Preeclampsia; [2]RBC: Red Blood Cells; [3]WBC: White Blood Cells; [4]AST: Aspartate Transaminase; [5]ALT: Alanine Transaminase; [6]gGT: gamma-Glutamyltransferase; [7]ALP: Alkaline Phosphatase; [8]CRP: C-Reactive Protein

Table S13. Neonatal results regarding Preeclampsia's severity Preeclampsia's onset (Early-Onset Preeclampsia subgroup, Late-Onset Preeclampsia subgroup and Controls). This table presents the unadjusted and adjusted p-values. Adjusted p-values are presented only for the parameters that differed statistically. The p-values are adjusted for the following covariates: Gestational Age, Birthweight, Length, Head Circumference, Apgar score at 1 and 5 minutes. P-values in bold indicate statistical significance at the level α=0.05.

NEONATES		Neonates born to Early-Onset PE[1] mothers (N=21)	Neonates born to Late-Onset PE mothers (N=13)	Neonates born to pregnant controls (N=34)	Adjusted P-Value (adjusted for covariates)			
					Across Subgroups	Early-Onset PE VS Late-Onset PE	Early-Onset PE VS Controls	Late-Onset PE VS Controls
	Platelet Count x10^9/L	195 (151-261)	234.5 (220.8-240.5)	275.5 (225.8-317)	**0.01**	0.81	**0.009**	0.13
	MPV[2] (fL)	7.4 (6.7-8.9)	7.5 (7-7.8)	9.3 (7.5-9.9)	**0.03**	0.97	**0.04**	0.13
	Platelet Count <100x10^9/L	0/21 (0%)	0/12 (0%)	0/34 (0%)	-	-	-	-
	Platelet Count <150x10^9/L	5/21 (23.8%)	0/12 (0%)	1/34 (2.9%)	**0.03**	1.00	0.20	1.00
CCTs[3]	PT[8] (seconds)	14.8 (13.4-16.8)	14.3 (13.9-15.3)	15 (13.5-15.8)	0.80	-	-	-
	APTT[9] (seconds)	45.7 (37.3-54)	39.2 (37.4-42.8)	43.8 (38.3-49.2)	0.30	-	-	-
	INR[10]	1.1 (0.99-1.21)	1.06 (1.05-1.11)	1.09 (0.99-1.15)	0.86	-	-	-
	Fibrinogen (mg/dL)	175 (155-281)	232 (203-253)	213 (172-287)	0.36	-	-	-
	D-Dimers (ng/mL)	2320 (1210-2900)	1390 (750-2300)	860 (592.3-1427.5)	**0.001**	0.97	**0.002**	**0.02**
INTEM[4]	CT[11] (seconds)	203 (189-254)	223 (208-239)	227.5 (191-247)	0.90	-	-	-
	CFT[12] (seconds)	99 (94-131)	88 (71-122)	74 (62.3-140.3)	**0.002**	**0.02**	**0.002**	1.00
	MCF[13] (mm)	48±5	54.4±6	54.7±9	**<0.001**	**0.002**	**<0.001**	1.00
	A-angle (° degrees)	71 (68-73)	72 (67-75)	75 (65.3-77)	0.09	-	-	-
	A10[14] (mm)	44 (41-46)	48 (44-53)	51 (41.3-58)	**<0.001**	**0.007**	**0.001**	1.00
	A30[14] (mm)	47.5±5	53.7±5.9	54.1±8.7	**<0.001**	**0.003**	**<0.001**	1.00
	MCE[15] (dynes/cm^2)	96 (79-105)	121 (110-131)	124 (94.3-166.5)	**<0.001**	**0.003**	**<0.001**	0.85
	LI60[16] (%)	92.6±2.6	90.6±3.9	92.2±3.4	0.21	-	-	-
	ML[17] (%)	12 (9-13)	14 (10-17)	10.5 (8-15)	0.21	-	-	-
	CFR[18] (° degrees)	73 (70-75)	74 (71-77)	76.5 (67-79)	0.14	-	-	-
	ACF[19] (mm)	42.7±5.1	46.9±6.4	48.5±9.3	**<0.001**	**0.006**	**<0.001**	1.00
EXTEM[5]	CT (seconds)	59 (50-65)	57 (53-65)	58 (51-72.8)	0.98	-	-	-
	CFT (seconds)	121.5 (102.8-158.8)	94 (78-131)	98.5 (78-149.8)	**0.02**	0.08	**0.02**	1.00
	MCF (mm)	49 (42-52)	55 (49-57)	53 (47-60.5)	**0.01**	0.05	**0.008**	1.00
	A-angle (° degrees)	67 (64-70)	71 (65-74)	70.5 (63-74)	0.10	-	-	-
	A10 (mm)	44 (34-45)	49 (42-53)	47 (39-55)	**0.02**	0.09	**0.02**	1.00
	A30 (mm)	48 (42-52)	55 (48-57)	52 (47-59.5)	**0.02**	0.07	**0.02**	1.00
	MCE (dynes/cm^2)	96 (73-105)	123 (95-133)	113.5 (88.3-150.8)	**0.009**	**0.047**	**0.008**	1.00
	LI60 (%)	90 (87-92)	90 (87-92)	92 (89.3-94.8)	0.055	-	-	-
	ML (%)	17 (13-25)	18 (12-29)	13 (8.3-16)	0.07	0.82	0.05	0.35
	CFR (° degrees)	70 (68-73)	74 (68-76)	73 (66.3-76)	0.24	-	-	-
	ACF (mm)	37.9±9.2	43±8.9	46.6±8.4	**0.002**	0.07	**0.002**	1.00
FIBTEM[6]	CT (seconds)	54 (51-63)	59 (53-88)	64 (51.5-74)	0.55	-	-	-
	CFT (seconds)	-	-	-	-	-	-	-
	MCF (mm)	11 (9-13)	14 (11-15)	15.5 (9.3-18)	**0.03**	0.21	**0.02**	0.96
	A-angle (° degrees)	66.6±8.1	65±4.3	69.7±7.6	0.42	-	-	-
	A10 (mm)	11 (8-12)	13 (10-14)	13 (9-16)	**0.02**	0.14	**0.01**	1.00
	A30 (mm)	11 (9-13)	14 (11-16)	16 (10-18.8)	**0.03**	0.21	**0.02**	1.00
	MCE (dynes/cm^2)	13 (10-15)	16 (12-18)	18.5 (10.3-22.8)	**0.02**	0.20	**0.008**	0.84
	LI60 (%)	96 (91-100)	99 (92-100)	98 (93.3-100)	0.74	-	-	-
	ML (%)	9 (2-13)	5 (2-14)	5.5 (0-10.8)	0.45	-	-	-

	CFR (° degrees)	69.6±7	66.5±4.2	71.3±5.8	0.24	-	-	-
	ACF (mm)	11 (9-12)	12 (11-15)	14.5 (9.3-17.8)	**0.03**	0.22	**0.02**	0.94
**APTEM[7]**	CT (seconds)	62 (50-69)	51 (49-58)	60 (47.3-80)	0.27	-	-	-
	CFT (seconds)	137.5 (112.8-164.3)	113 (82-157)	106.5 (80-146.8)	0.11	-	-	-
	MCF (mm)	46 (41-50)	51 (46-55)	52.5 (46-58.8)	**0.003**	0.06	**0.002**	1.00
	A-angle (° degrees)	65 (62-68)	70 (67-73)	70 (65-74)	0.11	-	-	-
	A10 (mm)	39 (37-43)	41 (40-48)	46 (37.5-53.8)	**0.006**	0.11	**0.003**	0.89
	A30 (mm)	44.3±9.7	49.2±8.7	52.5±8.6	**0.002**	0.09	**0.001**	1.00
	MCE (dynes/cm^2)	84 (68-101)	102 (86-120)	109.5 (85-143.5)	**0.002**	**0.049**	**0.001**	0.99
	LI60 (%)	90 (83-93)	90 (81-94)	93 (90-95)	0.07	-	-	-
	ML (%)	18.1±8.5	20.4±10	13.1±7.4	**0.02**	1.00	0.03	0.30
	CFR (° degrees)	69 (66.8-72.3)	74 (71-76)	73 (68-76)	0.23	-	-	-
	ACF (mm)	36.9±8.5	39.5±9.4	46.1±9.1	**<0.001**	0.09	**<0.001**	0.68

[1]PE: Preeclampsia; [2]MPV: Mean Platelet Volume; [3]CCTs: Conventional Coagulation Tests; [4]INTEM: Intrinsic Thromboelastometry; [5]EXTEM: Extrinsic Thromboelastometry; [6]FIBTEM: Fibrinogen Thromboelastometry; [7]APTEM: Aprotinin Thromboelastometry; [8]PT: Prothrombin Time; [9]APTT: Activated Partial Thromboplastin Time; [10]INR: International Normalized Ratio; [11]CT: Clotting Time; [12]CFT: Clot Formation Time; [13]MCF: Maximum Clot Firmness; [14]A10,30: Amplitude at 10, 30 minutes after Clotting Time (post CT); [15]MCE: Maximum Clot Elasticity; [16]LI60: Lysis Index at 60 minutes after Clotting Time (post CT); [17]ML: Maximum Lysis; [18]CFR: Clot Formation Rate; [19]ACF: Actual Clot Firmness

Table S14. All Spearman correlations between Platelet Count, Fibrinogen, D-dimers and Rotational Thromboelastometry (ROTEM) parameters regarding pregnant women. This table shows the correlations that were moderate ($0.40 \leq |\rho| \leq 0.59$), strong ($0.60 \leq |\rho| \leq 0.79$) or very strong ($0.80 \leq |\rho|$) and with a P-value ≤ 0.05.

**MODERATE CORRELATIONS**									
**Pregnant Women with PE[1]**					**Pregnant Controls**				
**Dependent Variable**	**Explanatory Variable**		**Spearman's "ρ"**	**P-Value**	**Dependent Variable**	**Explanatory Variable**		**Spearman's "ρ"**	**P-Value**
PLT[2]	INTEM[3]	CFT[7]	-0.48	0.006	PLT	INTEM	CFT	-0.45	0.002
		MCF[8]	0.49	0.005			-	-	-
		A-angle	0.46	0.01			A-angle	0.47	0.001
		A20[9]	0.51	0.003			-	-	-
		A30[9]	0.51	0.004			-	-	-
		MCE[10]	0.56	0.001			-	-	-
		CFR[11]	0.44	0.01			-	-	-
		ACF[12]	0.48	0.006			-	-	-
	EXTEM[4]	-	-	-		EXTEM	CFT	-0.43	0.003
		MCF	0.45	0.01			-	-	-
		A20	0.47	0.008			-	-	-
		A30	0.42	0.02			-	-	-
		LI30[13]	-0.51	0.004			-	-	-
		LI60[13]	-0.46	0.01			-	-	-
		MCE	0.54	0.002			-	-	-
		CFR	0.44	0.02			-	-	-
	FIBTEM[5]	MCF	0.53	0.002		FIBTEM	-	-	-
		A20	0.52	0.003			-	-	-
		A30	0.54	0.002			-	-	-
		MCE	0.56	0.001			-	-	-
		ACF	0.51	0.003			-	-	-
	APTEM[6]	CFT	-0.48	0.007		APTEM	-	-	-
		MCF	0.45	0.01			-	-	-
		A20	0.48	0.006			-	-	-
		A30	0.45	0.01			-	-	-
		MCE	0.50	0.004			-	-	-
Fibrinogen	INTEM	MCF	0.42	0.02	Fibrinogen	INTEM	MCF	0.46	0.002
		A20	0.43	0.02			A20	0.42	0.004
		A30	0.43	0.02			A30	0.43	0.004
		MCE	0.48	0.006			MCE	0.45	0.002
		ACF	0.42	0.02			ACF	0.50	<0.001
	EXTEM	CFT	-0.41	0.02		EXTEM	-	-	-
		A-angle	0.42	0.02			-	-	-
		MCE	0.40	0.03			-	-	-
		CFR	0.47	0.009			-	-	-
	FIBTEM	MCF	0.41	0.02		FIBTEM	MCF	0.51	<0.01
		A-angle	0.47	0.01			-	-	-
		A10[9]	0.40	0.03			A10	0.45	0.002
		-	-	-			A30	0.49	0.001
		MCE	0.42	0.02			MCE	0.51	<0.001
		CFR	0.53	0.003			-	-	-
		ACF	0.48	0.006			ACF	0.59	<0.001

	APTEM	MCF	0.43	0.02		APTEM	-	-	-
		A20	0.41	0.02			-	-	-
		A30	0.41	0.02			-	-	-
		MCE	0.45	0.01			-	-	-
		CFR	0.51	0.003			-	-	-
D-dimers	INTEM	CFT	0.44	0.01	D-dimers	INTEM	-	-	-
		A20	-0.42	0.02			-	-	-
		A30	-0.41	0.02			-	-	-
		MCE	-0.40	0.03			-	-	-
		CFR	-0.54	0.002			-	-	-
	EXTEM	CFT	0.46	0.009		EXTEM	-	-	-
	FIBTEM	MCF	-0.46	0.009		FIBTEM	-	-	-
		A20	-0.47	0.007			-	-	-
		A30	-0.46	0.01			-	-	-
		MCE	-0.42	0.02			-	-	-
		ACF	-0.42	0.02			-	-	-
	APTEM	CFT	0.50	0.004		APTEM	-	-	-
		MCF	-0.56	0.001			-	-	-
		A20	-0.57	0.001			-	-	-
		A30	-0.57	0.001			-	-	-
		MCE	-0.52	0.003			-	-	-
**STRONG AND VERY STRONG CORRELATIONS**									
**Pregnant Women with PE**					**Pregnant Controls**				
**Dependent Variable**	**Explanatory Variable**		**Spearman's "ρ"**	**P-Value**	**Dependent Variable**	**Explanatory Variable**		**Spearman's "ρ"**	**P-Value**
PLT	APTEM	LI30	-0.65	<0.001	PLT	APTEM	-	-	-
D-dimers	INTEM	A-angle	-0.60	<0.001	D-dimers	INTEM	-	-	-
	EXTEM	CT	0.71	<0.001		EXTEM	-	-	-
		MCF	-0.68	<0.001			-	-	-
		A-angle	-0.68	<0.001			-	-	-
		A20	-0.67	<0.001			-	-	-
		A30	-0.70	<0.001			-	-	-
		MCE	-0.66	<0.001			-	-	-
		CFR	-0.67	<0.001			-	-	-
		ACF	-0.72	<0.001			-	-	-

[1]PE: Preeclampsia; [2]PLT: Platelet Count; [3]INTEM: Intrinsic Thromboelastometry; [4]EXTEM: Extrinsic Thromboelastometry; [5]FIBTEM: Fibrinogen Thromboelastometry; [6]APTEM: Aprotinin Thromboelastometry; [7]CFT: Clot Formation Time; [8]MCF: Maximum Clot Firmness; [9]A10,20,30: Amplitude at 10, 20, 30 minutes after Clotting Time (post CT); [10]MCE: Maximum Clot Elasticity; [11]CFR: Clot Formation Rate; [12]ACF: Actual Clot Firmness; [13]LI30,60: Lysis Index at 30, 60 minutes after Clotting Time (post CT)

Table S15. All Spearman correlations between Platelet Count, Fibrinogen and Rotational Thromboelastometry (ROTEM) parameters regarding neonates. This table shows the correlations that were moderate (0.40 ≤ |ρ| ≤ 0.59), strong (0.60 ≤ |ρ| ≤ 0.79) or very strong (0.80 ≤ |ρ|) and with a P-value ≤0.05.

**MODERATE CORRELATIONS**									
**Neonates born to women with PE[1]**					**Neonates born to pregnant controls**				
**Dependent Variable**	**Explanatory Variable**		**Spearman's "ρ"**	**P-Value**	**Dependent Variable**	**Explanatory Variable**		**Spearman's "ρ"**	**P-Value**
PLT[2]	INTEM[3]	-	-	-	PLT	INTEM	CFT[8]	-0.50	<0.001
		-	-	-			MCF[9]	0.52	<0.001
		-	-	-			A-angle	0.47	0.001
		-	-	-			A20[10]	0.53	<0.001
		-	-	-			A30[10]	0.49	<0.001
		-	-	-			LI30	-0.43	0.003
		-	-	-			MCE[11]	0.52	<0.001
		-	-	-			CFR[12]	0.46	0.001
	EXTEM[4]	-	-	-		EXTEM	CFT	-0.52	<0.001
		-	-	-			MCF	0.45	0.001
		-	-	-			A-angle	0.54	<0.001
		-	-	-			A20	0.45	0.001
		-	-	-			A30	0.40	0.005
		-	-	-			LI30	-0.43	0.002
		-	-	-			MCE	0.45	0.002
		-	-	-			CFR	0.52	<0.001
	FIBTEM[5]	-	-	-		FIBTEM	LI30	-0.57	<0.001
	APTEM[6]	-	-	-		APTEM	CFT	-0.57	<0.001
		-	-	-			MCF	0.49	<0.001
		-	-	-			A-angle	0.52	<0.001
		-	-	-			A20	0.49	<0.001
		-	-	-			A30	0.45	0.002
		-	-	-			LI30	-0.41	0.004
		-	-	-			LI60[7]	-0.44	0.002
		-	-	-			MCE	0.49	<0.001
		-	-	-			CFR	0.49	<0.001
Fibrinogen	EXTEM	-	-	-	Fibrinogen	EXTEM	MCF	0.44	0.003
		-	-	-			A20	0.46	0.001
		-	-	-			A30	0.46	0.001
		-	-	-			MCE	0.44	0.002
	APTEM	A-angle	0.40	0.02		APTEM	-	-	-
		LI30[7]	0.50	0.002			-	-	-

**STRONG AND VERY STRONG CORRELATIONS**									
**Neonates born to women with PE**					**Neonates born to pregnant controls**				
**Dependent Variable**	**Explanatory Variable**		**Spearman's "ρ"**	**P-Value**	**Dependent Variable**	**Explanatory Variable**		**Spearman's "ρ"**	**P-Value**
Fibrinogen	FIBTEM	A-angle	-0.71	0.02	Fibrinogen	FIBTEM	-	-	-

[1]PE: Preeclampsia; [2]PLT: Platelet Count; [3]INTEM: Intrinsic Thromboelastometry; [4]EXTEM: Extrinsic Thromboelastometry; [5]FIBTEM: Fibrinogen Thromboelastometry; [6]APTEM: Aprotinin Thromboelastometry; [7]LI30,60: Lysis Index at 30, 60 minutes after Clotting Time (post CT); [8]CFT: Clot Formation Time; [9]MCF: Maximum Clot Firmness; [10]A10,20,30: Amplitude at 10, 20, 30 minutes after Clotting Time (post CT); [11]MCE: Maximum Clot Elasticity; [12]CFR: Clot Formation Rate

Table S16. Regression models for strong and very strong correlations regarding pregnant women with Preeclampsia. The selection of the model was based on the statistical significance of the p-values of the intercept and the coefficients and the adjusted R^2. For each correlation the selected model is highlighted in red. P-values in bold indicate statistical significance at the level α=0.05.

Dependent Variable	Independent Variable	Type of Regression	Regression Details									
LI30[1] APTEM[2]	PLT[3]	LINEAR	Intercept (b0)	b0 P-Value	b1	b1 P-Value	Overall P-value	Adjusted R^2				
			100.27	**<0.001**	-0.002	**<0.001**	**<0.001**	0.41				
		QUADRATIC	Intercept (b0)	b0 P-Value	b1	b1 P-Value	b2	b2 P-Value	Overall P-value	Adjusted R^2		
			99.73	**<0.001**	0.004	**<0.001**	-0.00001	**<0.001**	**<0.001**	0.88		
		CUBIC	Intercept (b0)	b0 P-Value	b1	b1 P-Value	b2	b2 P-Value	b3	b3 P-Value	Overall P-value	Adjusted R^2
			100.13	**<0.001**	-0.004	**<0.001**	0.00003	**<0.001**	-0.00000007	**<0.001**	**<0.001**	**0.99**
A-angle INTEM[4]	D-dimers	LINEAR	Intercept (b0)	b0 P-Value	b1	b1 P-Value	Overall P-value	Adjusted R^2				
			81.60	**<0.001**	-0.002	**<0.001**	**<0.001**	0.34				
		QUADRATIC	Intercept (b0)	b0 P-Value	b1	b1 P-Value	b2	b2 P-Value	Overall P-value	Adjusted R^2		
			78.36	**<0.001**	0.0008	0.67	-0.0000004	0.13	**<0.001**	0.42		
		CUBIC	Intercept (b0)	b0 P-Value	b1	b1 P-Value	b2	b2 P-Value	b3	b3 P-Value	Overall P-value	Adjusted R^2
			81.73	**<0.001**	-0.004	0.37	0.000002	0.36	-0.0000000002	0.25	**0.001**	0.38
CT[5] EXTEM[6]	D-dimers	LINEAR	Intercept (b0)	b0 P-Value	b1	b1 P-Value	Overall P-value	Adjusted R^2				
			2.02	0.90	0.03	**<0.001**	**<0.001**	0.49				
		QUADRATIC	Intercept (b0)	b0 P-Value	b1	b1 P-Value	b2	b2 P-Value	Overall P-value	Adjusted R^2		
			103.33	**<0.001**	-0.05	**0.003**	0.00001	**<0.001**	**<0.001**	0.74		
		CUBIC	Intercept (b0)	b0 P-Value	b1	b1 P-Value	b2	b2 P-Value	b3	b3 P-Value	Overall P-value	Adjusted R^2
			-0.83	0.97	0.10	**<0.001**	-0.00005	**<0.001**	0.000000006	**<0.001**	**<0.001**	**0.89**
MCF[7] EXTEM	D-dimers	LINEAR	Intercept (b0)	b0 P-Value	b1	b1 P-Value	Overall P-value	Adjusted R^2				
			79.53	**<0.001**	-0.005	**<0.001**	**<0.001**	**0.45**				
		QUADRATIC	Intercept (b0)	b0 P-Value	b1	b1 P-Value	b2	b2 P-Value	Overall P-value	Adjusted R^2		
			73.90	**<0.001**	-0.0006	0.88	-0.0000007	0.25	**<0.001**	**0.45**		
		CUBIC	Intercept (b0)	b0 P-Value	b1	b1 P-Value	b2	b2 P-Value	b3	b3 P-Value	Overall P-value	Adjusted R^2
			76.27	**<0.001**	-0.004	0.71	0.0000007	0.86	-0.0000000001	0.73	**<0.001**	0.44
A-angle EXTEM	D-dimers	LINEAR	Intercept (b0)	b0 P-Value	b1	b1 P-Value	Overall P-value	Adjusted R^2				
			85.42	**<0.001**	-0.004	**<0.001**	**<0.001**	0.45				
		QUADRATIC	Intercept (b0)	b0 P-Value	b1	b1 P-Value	b2	b2 P-Value	Overall P-value	Adjusted R^2		
			76.43	**<0.001**	0.003	0.30	-0.000001	**0.01**	**<0.001**	0.55		
		CUBIC	Intercept (b0)	b0 P-Value	b1	b1 P-Value	b2	b2 P-Value	b3	b3 P-Value	Overall P-value	Adjusted R^2
			90.98	**<0.001**	-0.02	**0.005**	0.000007	**0.003**	-0.0000000009	**<0.001**	**<0.001**	**0.70**
A20[8] EXTEM	D-dimers	LINEAR	Intercept (b0)	b0 P-Value	b1	b1 P-Value	Overall P-value	Adjusted R^2				
			79.72	**<0.001**	-0.006	**<0.001**	**<0.001**	**0.43**				
		QUADRATIC	Intercept (b0)	b0 P-Value	b1	b1 P-Value	b2	b2 P-Value	Overall P-value	Adjusted R^2		
			71.25	**<0.001**	0.0009	0.86	0.000001	0.15	**<0.001**	0.46		
		CUBIC	Intercept (b0)	b0 P-Value	b1	b1 P-Value	b2	b2 P-Value	b3	b3 P-Value	Overall P-value	Adjusted R^2
			75.56	**<0.001**	-0.006	0.67	0.000001	0.75	-0.0000000003	0.59	**<0.001**	0.44
A30[8] EXTEM	D-dimers	LINEAR	Intercept (b0)	b0 P-Value	b1	b1 P-Value	Overall P-value	Adjusted R^2				
			80.07	**<0.001**	-0.006	**<0.001**	**<0.001**	0.47				
		QUADRATIC	Intercept (b0)	b0 P-Value	b1	b1 P-Value	b2	b2 P-Value	Overall P-value	Adjusted R^2		
			73.94	**<0.001**	-0.0008	0.86	-0.0000007	0.24	**<0.001**	0.48		
		CUBIC	Intercept (b0)	b0 P-Value	b1	b1 P-Value	b2	b2 P-Value	b3	b3 P-Value	Overall P-value	Adjusted R^2
			76.62	**<0.001**	-0.005	0.68	0.0000008	0.85	-0.0000000002	0.71	**<0.001**	0.46
MCE[9] EXTEM	D-dimers	LINEAR	Intercept (b0)	b0 P-Value	b1	b1 P-Value	Overall P-value	Adjusted R^2				
			310.76	**<0.001**	-0.03	**<0.001**	**<0.001**	**0.41**				
		QUADRATIC	Intercept (b0)	b0 P-Value	b1	b1 P-Value	b2	b2 P-Value	Overall P-value	Adjusted R^2		
			313.18	**<0.001**	-0.04	0.22	0.0000003	0.95	**<0.001**	0.39		
		CUBIC	Intercept (b0)	b0 P-Value	b1	b1 P-Value	b2	b2 P-Value	b3	b3 P-Value	Overall P-value	Adjusted R^2
			360.91	**<0.001**	-0.11	0.18	0.00003	0.34	-0.000000003	0.34	**<0.001**	0.39
CFR[10] EXTEM	D-dimers	LINEAR	Intercept (b0)	b0 P-Value	b1	b1 P-Value	Overall P-value	Adjusted R^2				
			84.68	**<0.001**	-0.003	**<0.001**	**<0.001**	0.42				
		QUADRATIC	Intercept (b0)	b0 P-Value	b1	b1 P-Value	b2	b2 P-Value	Overall P-value	Adjusted R^2		
			79.69	**<0.001**	0.0006	0.80	-0.0000006	0.10	**<0.001**	0.46		
		CUBIC	Intercept (b0)	b0 P-Value	b1	b1 P-Value	b2	b2 P-Value	b3	b3 P-Value	Overall P-value	Adjusted R^2
			90.41	**<0.001**	-0.02	**0.01**	0.000005	**0.01**	-0.0000000006	**0.006**	**<0.001**	**0.59**
ACF[11] EXTEM	D-dimers	LINEAR	Intercept (b0)	b0 P-Value	b1	b1 P-Value	Overall P-value	Adjusted R^2				
			71.35	**<0.001**	-0.005	**<0.001**	**<0.001**	**0.50**				

		QUADRATIC	Intercept (b0)	b0 P-Value	b1	b1 P-Value	b2	b2 P-Value	Overall P-value	Adjusted R^2		
			73.61	**<0.001**	-0.006	0.06	0.0000003	0.57	**<0.001**	0.49		
		CUBIC	Intercept (b0)	b0 P-Value	b1	b1 P-Value	b2	b2 P-Value	b3	b3 P-Value	Overall P-value	Adjusted R^2
			70.80	**<0.001**	-0.002	0.80	-0.000001	0.68	0.0000000002	0.62	**<0.001**	0.47

[1]LI30: Lysis Index at 30 minutes after Clotting Time (post CT); [2]APTEM: Aprotinin Thromboelastometry; [3]PLT: Platelets; [4]INTEM: intrinsic Thromboelastometry; [5]CT: Clotting Timel; [6]EXTEM: Extrinsic Thromboelastometry; [7]MCF: maximum Clot Firmness; [8]A20,30: Amplitude at 20,30 minutes after Clotting Time (post CT); [9]MCE: Maximum Clot Elasticity; [10]CFR: Clot Formation Rate; [11]ACF: Actual Clot Firmness;

Table S17. All logistic regressions with P-value ≤ 0.05 regarding pregnant women with Preeclampsia and neonates born to pregnant women with Preeclampsia.

**PREGNANT WOMEN WITH PREECLAMPSIA**							
**Dependent Variable**	**Independent**	**P-Value**	**OR[1]**	**OR 95% CI[2]**	**Residual Deviance**	**AIC[3]**	**Hosmer–Lemeshow test**
Severe Preeclampsia	CFT[4] INTEM[5]	**0.03**	1.02	1.003-1.04	57.8	61.8	0.10
	CFT EXTEM[6]	**0.03**	1.01	1.001-1.02	58.1	62.1	**0.02**
Maternal persistent thrombocytopenia (after delivery)	CFT INTEM	**0.02**	1.03	1.004-1.05	20.0	24.0	0.41
	MCF[7] INTEM	**0.045**	0.87	**0.76-1.007**	21.8	25.8	0.27
	A10[8] INTEM	**0.02**	0.88	0.78-0.98	20.0	24.0	0.35
	CFT EXTEM	**0.01**	1.02	1.003-1.03	19.8	23.8	0.23
	A-angle EXTEM	**0.04**	0.89	0.80-0.99	21.0	25.0	0.20
	A10 EXTEM	**0.03**	0.93	0.86-0.99	21.1	25.1	0.21
	MCE[9] EXTEM	**0.04**	0.98	0.96-0.99	20.5	24.5	0.32
	CFR[10] EXTEM	**0.02**	0.83	0.72-0.97	19.1	23.1	0.31
	CFT APTEM[11]	**0.02**	1.02	1.004-1.04	19.7	23.7	0.30
	MCF APTEM	**0.03**	0.90	0.81-0.99	21.0	25.0	0.19
	A10 APTEM	**0.02**	0.89	0.80-0.98	19.2	23.2	0.31
	MCE APTEM	**0.03**	0.98	0.96-0.99	18.8	22.8	0.46
Maternal PLT < 150 $x10^9$/L	CFT INTEM	**0.02**	1.02	1.01-1.04	62.6	66.6	0.81
	A-angle INTEM	**0.044**	0.87	0.75-0.99	65.5	69.5	0.22
	LI60[12] INTEM	**0.03**	1.55	1.05-2.29	58.3	62.3	0.56
	ML[13] INTEM	**0.03**	0.80	0.65-0.98	64.1	68.1	0.20
	CFT EXTEM	**0.01**	1.02	1.01-1.03	61.2	65.2	0.11
	MCF EXTEM	**0.008**	0.89	0.81-0.97	59.3	63.3	0.40
	A-angle EXTEM	**0.02**	0.89	0.80-0.98	58.6	62.6	0.13
	A30 EXTEM	**0.008**	0.89	0.80-0.96	58.9	62.9	0.42
	MCE EXTEM	**0.008**	0.99	0.97-0.99	61.1	65.1	0.16
	CFR EXTEM	**0.009**	0.86	0.77-0.96	58.2	62.2	0.12
	ACF[14] EXTEM	**0.01**	0.90	0.84-0.98	61.3	65.3	0.37
	CFT APTEM	**0.03**	1.01	1.002-1.03	64.7	68.7	**0.046**
Maternal PLT transfusion	CFT INTEM	**0.03**	1.02	1.001-1.04	32.6	36.6	0.74
	CFT EXTEM	**0.02**	1.01	1.001-1.02	32.5	36.5	0.20
	CFT APTEM	**0.03**	1.02	1.001-1.03	32.7	36.7	0.21
**NEONATES BORN TO WOMEN WITH PREECLAMPSIA**							
**Dependent Variable**	**Independent**	**P-Value**	**OR**	**OR 95% CI**	**Residual Deviance**	**AIC**	**Hosmer–Lemeshow test**
Neonatal PLT < 150 $x10^9$/L	Neonatal MCF EXTEM	**0.03**	0.88	0.79-0.99	34.1	38.1	**0.02**
	Neonatal A30 EXTEM	**0.03**	0.88	0.79-0.99	34.3	38.3	**0.02**
	Neonatal ACF EXTEM	**0.046**	0.93	0.86-0.99	37.8	41.8	**0.048**
	Neonatal CFR APTEM	**0.049**	0.92	**0.85-1.001**	37.9	41.9	0.60

[1]OR: Odds Ratio; [2]CI: Confidence Interval; [3]AIC: Akaike Information Criterion; [4]CFT: Clot Formation Time; [5]INTEM: Intrinsic Thromboelastometry; [6]EXTEM: Extrinsic Thromboelastometry; [7]MCF: Maximum Clot Firmness; [8]A10,20,30: Amplitude at 10, 20, 30 minutes after Clotting Time (post CT); [9]MCE: Maximum Clot Elasticity; [10]CFR: Clot Formation Rate; [11]APTEM: Aprotinin Thromboelastometry; [12]LI30,60: Lysis Index at 30, 60 minutes after Clotting Time (post CT); [13]ML: Maximum Lysis; [14]ACF: Actual Clot Firmness

Table S18. All variables for which a Receiver Operating Characteristic (ROC) curve was created based on the logistic regression's results. P-values in bold indicate statistical significance at the level α=0.05.

			AUC[1]	AUC 95% CI[2]	Smooth AUC	Smooth AUC 95% CI	Optimal Cutoff Point	Sensitivity at Cutoff Point	Specificity at Cutoff Point
Preeclampsia's severity	Maternal CFT[3] INTEM[4]		0.48	0.26-0.70	0.45	0.26-0.67	-	-	-
			**AUC**	**AUC 95% CI**	**Smooth AUC**	**Smooth AUC 95% CI**	**Optimal Cutoff Point**	**Sensitivity at Cutoff Point**	**Specificity at Cutoff Point**
Maternal persistent thrombocytopenia (up to 7 days after delivery)	INTEM	Maternal CFT INTEM	0.82	0.55-0.99	0.79	0.75-0.84	107 seconds	0.66	0.95
		Maternal A10[7] INTEM	0.83	0.58-1.00	0.82	0.76-0.87	53.5 mm	0.67	0.95
	EXTEM[5]	Maternal CFT EXTEM	0.95	0.90-0.99	0.95	0.90-0.99	117 seconds	1.00	0.95
		Maternal A-angle EXTEM	0.95	0.89-0.99	0.95	0.90-0.98	72 mm	0.99	0.94
		Maternal A10 EXTEM	0.95	0.90-0.99	0.94	0.90-0.98	53.5 mm	0.98	0.93
		Maternal MCE[8] EXTEM	0.90	0.81-0.99	0.89	0.82-0.95	193 dynes/cm^2	1.00	0.84
		Maternal CFR[9] EXTEM	0.95	0.89-0.99	0.95	0.89-0.98	73°	1.00	0.93
	APTEM[6]	Maternal CFT APTEM	0.94	0.87-0.99	0.93	0.86-0.98	131 seconds	1.00	0.92
		Maternal MCF[10] APTEM	0.92	0.85-0.98	0.92	0.85-0.96	61mm	1.00	0.89
		Maternal A10 APTEM	0.94	0.88-0.99	0.93	0.88-0.98	49 mm	1.00	0.90
		Maternal MCE APTEM	0.92	0.85-0.98	0.91	0.85-0.96	156.5 dynes/cm^2	1.00	0.89
	**Bootstrap Tests**				**P-Value**				
	ROC[11] Maternal CFT INTEM vs ROC Maternal A10 INTEM				0.28				
	ROC Maternal CFT EXTEM vs ROC Maternal A-angle EXTEM				0.27				
	ROC Maternal CFT EXTEM vs ROC Maternal A10 EXTEM				0.31				
	ROC Maternal CFT EXTEM vs ROC Maternal MCE EXTEM				**0.02**				
	ROC Maternal CFT EXTEM vs ROC Maternal CFR EXTEM				0.47				
	ROC Maternal A-angle EXTEM vs ROC Maternal A10 EXTEM				0.18				
	ROC Maternal A-angle EXTEM vs ROC Maternal MCE EXTEM				0.06				
	ROC Maternal A-angle EXTEM vs ROC Maternal CFR EXTEM				0.34				
	ROC Maternal A10 EXTEM vs ROC Maternal MCE EXTEM				**0.02**				
	ROC Maternal A10 EXTEM vs ROC Maternal CFR EXTEM				0.33				
	ROC Maternal CFR EXTEM vs ROC Maternal MCE EXTEM				**0.03**				
	ROC Maternal CFT APTEM vs ROC Maternal MCF[13] APTEM				0.56				
	ROC Maternal CFT APTEM vs ROC Maternal A10 APTEM				0.65				
	ROC Maternal CFT APTEM vs ROC Maternal MCE APTEM				0.58				
	ROC Maternal MCF APTEM vs ROC Maternal A10 APTEM				0.17				
	ROC Maternal MCF APTEM vs ROC Maternal MCE APTEM				1.00				
	ROC Maternal MCE APTEM vs ROC Maternal A10 APTEM				0.16				
			**AUC**	**AUC 95% CI**	**Smooth AUC**	**Smooth AUC 95% CI**	**Optimal Cutoff Point**	**Sensitivity at Cutoff Point**	**Specificity at Cutoff Point**
Maternal PLT transfusion (at delivery or up to 7 days after delivery)	INTEM	Maternal CFT INTEM	0.69	0.33-0.96	0.68	0.40-0.94	95.5 seconds	0.60	0.93
	EXTEM	Maternal CFT EXTEM	0.81	0.52-0.99	0.80	0.47-0.97	108 seconds	0.80	0.94
	APTEM	Maternal CFT APTEM	0.77	0.44-0.97	0.77	0.43-0.96	124 seconds	0.80	0.92
	**Bootstrap Tests**				**P-Value**				
	ROC Maternal CFT INTEM vs ROC Maternal CFT EXTEM				0.13				
	ROC Maternal CFT INTEM vs ROC Maternal CFT APTEM				0.29				
	ROC Maternal CFT EXTEM vs ROC Maternal CFT APTEM				0.23				

[1]AUC: Area Under the Curve; [2]CI: Confidence Interval; [3]CFT: Clot Formation Time; [4]INTEM: Intrinsic Thromboelastometry; [5]EXTEM: Extrinsic Thromboelastometry; [6]APTEM: Aprotinin Thromboelastometry; [7]A10: Amplitude at 10 minutes after Clotting Time (post CT); [8]MCE: Maximum Clot Elasticity; [9]CFR: Clot Formation Rate; [10]MCF: Maximum Clot Firmness; [11]ROC: Receiver Operating Characteristic
